# Discovery of potent and specific inhibitors targeting the active site of MMP-9 from the engineered SPINK2 library

**DOI:** 10.1371/journal.pone.0244656

**Published:** 2020-12-29

**Authors:** Hidenori Yano, Daisuke Nishimiya, Yoshirou Kawaguchi, Masakazu Tamura, Ryuji Hashimoto

**Affiliations:** 1 Modality Research Laboratories, Biologics Division, Daiichi Sankyo Co., Ltd., Tokyo, Japan; 2 Graduate School of Life and Environmental Sciences, University of Tsukuba, Ibaraki, Japan; Cardiff University, UNITED KINGDOM

## Abstract

Matrix metalloproteinases (MMPs) contribute to many physiological and pathological phenomena via the proteolysis of extracellular matrix components. Specific blocking of the active site of each MMP sheds light on its particular role. However, it remains difficult to acquire an active-site inhibitor with high specificity for only the target MMP due to the highly conserved structure around the active site of MMPs. Recently, we reported that potent and specific inhibitors of serine proteases were obtained from our proprietary engineered serine protease inhibitor Kazal type 2 (SPINK2) library. In this research, using this library, we succeeded in obtaining potent and specific MMP-9 inhibitors. The obtained inhibitors bound to the active site of MMP-9 and inhibited MMP-9 with low nanomolar *K*_*i*_ values. The inhibitors did not cross-react with other MMPs that we tested. Further analysis using MMP-9 mutants demonstrated that the inhibitors recognize not only the residues around the conserved active site of MMP-9 but also different and unique residues in exosites that are distant from each other. This unique recognition manner, which can be achieved by the large interface provided by engineered SPINK2, may contribute to the generation of specific active-site inhibitors of MMPs.

## Introduction

Matrix metalloproteinases (MMPs) contribute to the degradation of extracellular matrix (ECM) proteins (*e*.*g*., collagen, elastin, fibronectin, and laminin) and the processing of pro-inflammatory cytokines (*e*.*g*., IL-1β) and chemokines (*e*.*g*., IL-8/CXCL8). Through these proteolytic functions, MMPs play crucial roles in many physiological functions related to cell growth, differentiation, migration, and apoptosis. It has also been reported that aberrant expression or activation of MMPs may cause pathological conditions such as inflammation [1] and tumor metastasis [2]. Twenty-three MMP proteins have been reported in humans to date [3,4] and several MMPs are known to be upregulated in diseases involving abnormal degradation of the extracellular matrix, such as inflammatory bowel disease [3]. To further advance MMP-related research, it is highly desirable to obtain molecules that specifically inhibit the enzymatic activity of a target MMP. Revealing the roles of individual MMPs in physiological and pathological conditions using specific inhibitors can contribute to the generation of therapeutics targeting MMPs.

A catalytic zinc ion, which is chelated by three histidine residues, and a catalytic glutamate residue are essential for the proteolytic activity of MMPs and constitute their active site. The sequence motif of this active site of MMPs, HEXGHXXGXXH, is highly conserved within the MMP family. MMPs are translated and secreted as the pro-form (pro-MMPs) without proteolytic activity. Both the active site and the surrounding region of the active site, the so-called active-site cleft, are completely masked by the pro-peptide in pro-MMPs, interfering with the interaction between the active site and the substrate [5]. Pro-MMP is activated by enzymatic digestion (*e*.*g*., by plasmin, trypsin, and other MMPs) [6,7] or non-proteolytic modification [8–10] that causes removal of the pro-peptide from the active-site cleft. MMPs share a consensus cleavage motif, proline at P3 and a hydrophobic residue at P1′, which results from high sequence homology of the active-site cleft [11,12]. Each MMP shows some substrate specificity towards macromolecular substrates [4]. This is because not only the active site but also a secondary binding site away from the active site, called an exosite, is involved in substrate recognition by MMPs. For example, some domains in MMPs are essential for the degradation of collagen: the hemopexin (HPX) domain fused to the catalytic domain by a linker in MMP-1, -8, -13, and -14; and the fibronectin (Fn)-like domain inserted in the catalytic domain in MMP-2 and -9 [13–15]. MMPs utilize such domains that exhibit some diversity among MMP family members as exosites to recognize macromolecular substrates, facilitating the maintenance of an appropriate distance and direction between the cleavage site and the active site; this in turn contributes to the specific degradation of macromolecular substrates.

The proteolytic activities of MMPs are completely inhibited by molecules that bind to their active site. Many chemical compounds that interact with part of the active site have been reported as active-site inhibitors of MMPs. Most of these active-site chemical inhibitors show high potency by chelating the catalytic zinc ion; however, they also show broad inhibitory activity across the whole family of MMPs due to recognition of the highly homologous active-site cleft. Such non-specific MMP inhibitors (*e*.*g*., hydroxamate-based inhibitors) achieved promising therapeutic effects in several preclinical studies for cancer; however, in clinical trials, these inhibitors caused serious side effects such as musculoskeletal pain and inflammation due to the broad inhibition of MMPs, so all of these trials failed [3]. Therefore, specific active-site inhibitors of MMPs are strongly desired from a clinical perspective. Protein-based active-site inhibitors that have the potential to recognize both the active site and the exosite in a manner similar to that of macromolecular substrates were also reported. Anti-MMP-9 antibodies, SDS3 and SDS4, showed high potency with active-site binding but also inhibited MMP-2, the sequence of which is the most similar to that of MMP-9 among the MMP family [16]. Several attempts to generate specific active-site inhibitors from broadly inhibitory proteins by protein engineering were also reported. Endogenous tissue inhibitors of metalloproteinases (TIMPs) strongly but non-selectively inhibit MMPs by coordinating the catalytic zinc ion [17]. Mutations to TIMP residues that interact with the exosite of MMPs improved the specificity to MMPs [18–20]. However, engineered TIMPs that could inhibit only a single MMP have not been reported. Other attempts have been made to target exosite domains, such as the HPX domain, which are less conserved. Several exosite inhibitors abrogated cancer cell migration through specific prevention of the association of pro-MMP-9 with both α4β1 integrin and CD44 without modulating the catalytic activity of MMP-9 [21–23]. Although exosite recognition could contribute to improving the specificity of active-site inhibitors, it remains difficult to obtain specific active-site inhibitors against a single MMP.

Recently, we reported that potent and specific inhibitors of serine proteases were obtained from our proprietary technology, namely, the engineered serine protease inhibitor Kazal type 2 (SPINK2) library [24]. Here, using this library, we attempted to obtain a specific active-site inhibitor of MMP-9, which is involved in the degradation of ECM proteins including type IV collagen, gelatin, and elastin [10]. MMP-9 breaks down the physical barrier formed by ECM, promoting cancer metastasis and inflammation [1,25,26]. Furthermore, aberrant MMP-9 activity causes blood-brain barrier disruption which can involve in numerous neurodegenerative diseases such as multiple sclerosis, stroke, epilepsy, brain tumors, and Alzheimer’s disease [27,28]. Recent studies have reported the promising efficacy of MMP-9 inhibitors in several pathological in vivo models [29–32], it is also required to create MMP-9 inhibitors with higher potency and selectivity [26]. We demonstrate that several inhibitors obtained from our library strongly inhibit the proteolytic activity of MMP-9 and show high specificity to only MMP-9, while not inhibiting the other 12 MMPs. Several assays using MMP-9 mutants suggested that these inhibitors recognized not only the highly conserved active site but also the specific exosite of MMP-9 to achieve the high specificity. These specific MMP-9 inhibitors discovered from the engineered SPINK2 library may contribute to clarifying the roles of MMP-9 under physiological and pathological conditions and to the development of therapeutics for MMP-9-related diseases.

## Results

### MMP-9 inhibitors were isolated from the engineered SPINK2 phage display library

Prior to the screening of inhibitors against MMP-9 from the engineered SPINK2 phage library, we designed a recombinant pro-MMP-9 construct and examined the method of its activation to prepare highly qualified bait proteins for phage panning. First, we investigated which domains were required for the bait protein. MMP-9 has several functional domains: pro-peptide domain, catalytic domain, Fn-like domain, and HPX domain [1]. As the Fn-like domain directly contacts the catalytic domain [5], it is preferable to use a bait protein that retains the Fn-like domain in order to obtain inhibitors recognizing the native structure of MMP-9. On the other hand, the HPX domain is connected to the catalytic domain by a long and flexible linker region [33]; thus, deletion of the HPX domain would not affect the surface exposure of the catalytic domain. Hence, we prepared a pro-MMP-9 catalytic domain with an Fn-like domain and without an HPX domain (pro-MMP-9_Cat) for subsequent experiments. In general, proteases labeled by random biotinylation using chemical reagents can be inactive due to blocking of the active-site cleft, so we prepared biotinylated pro-MMP-9_Cat with a site-specific biotin acceptor peptide (BAP) [34] using biotin-protein ligase BirA. Finally, to obtain homogeneous active MMP-9, we examined several methods to activate pro-MMP-9 using MMP-3 [6], trypsin [7], or 4-aminophenylmercuric acetate (APMA) [8,9]. Trypsin could activate pro-MMP-9_Cat, but it also digested various sites in MMP-9 and cleaved off its C-terminal tag (S9 Fig). Compared with the method using APMA, MMP-9 activated by MMP-3 showed higher proteolytic activity as previously reported [35]. In addition, fewer extra bands including those representing inactivated pro-MMP-9 and degraded MMP-9 were detected in SDS-PAGE, indicating that MMP-9 activated by MMP-3 was more homogeneous. Thus, we prepared biotinylated active MMP-9_Cat-BAP as the highly qualified bait protein by the activation method using MMP-3 (S1A Fig).

Then, we carried out phage panning against biotinylated active MMP-9_Cat-BAP using the engineered SPINK2 phage library [24]. After three rounds of panning, we screened approximately 2,000 clones as thioredoxin tag-fused proteins expressed in *E*. *coli* by MMP-9 inhibitory assay using FRET peptide substrate. As a result, we obtained more than 380 hit clones. One hundred and thirty unique inhibitors were identified by DNA sequencing, indicating that the hit rate of unique inhibitors in this screening was 6.5%. Fourteen inhibitors with IC_50_ <10 nM were obtained in the MMP-9 inhibitory assay, and we finally selected four inhibitors: M91002, M91005, M91011, and M91012. Comparing the loop sequences of their inhibitors (S2D Fig) with previously reported MMP-9 substrate sequences [11,36] and the information in the MEROPS database, no substrate sequence motifs of MMP-9 were included in their inhibitors. We purified the selected inhibitors by removing the thioredoxin tag, followed by size-exclusion chromatography for further evaluation.

### Engineered SPINK2-derived inhibitors show potent and specific MMP-9 inhibitory activity

**Fig 1:**
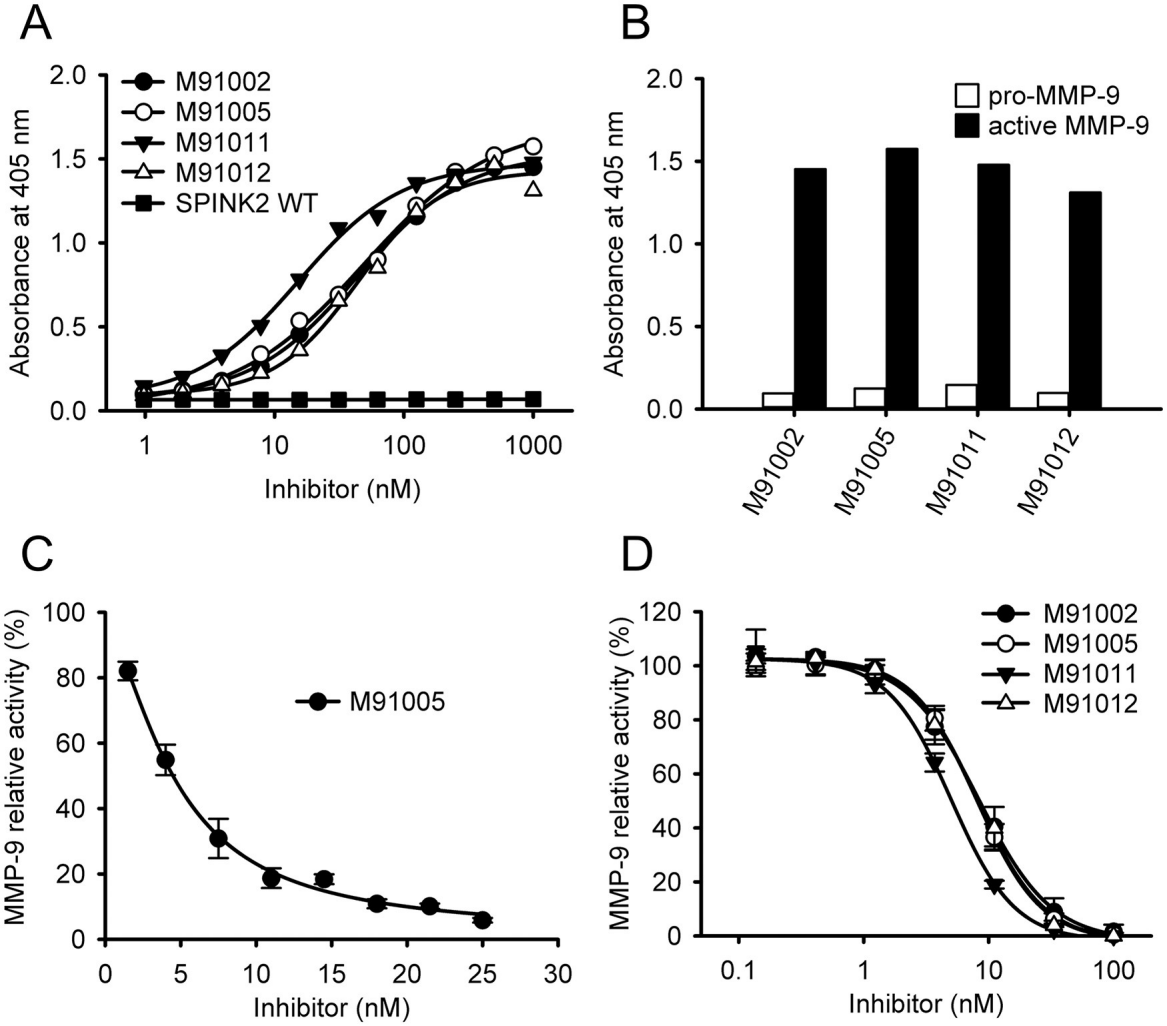
Binding and inhibitory activity of engineered SPINK2-derived inhibitors. (A) The binding activity to active MMP-9 was measured by ELISA. Various concentrations of engineered SPINK2-derived inhibitors or wild-type SPINK2 (1–1,000 nM) were added to the biotinylated active MMP-9_Cat-Avi (50 nM)-coated plate, and then inhibitors bound to active MMP-9 were detected by HRP-conjugated anti-S tag antibody. (B) The binding activities to pro- and active MMP-9 were measured by ELISA. Biotinylated pro-MMP-9_Cat-Avi or biotinylated active MMP-9_Cat-Avi (50 nM each) was coated on the plate, to which inhibitors (1 μM) were then added. The inhibitors bound to MMP-9 were detected by HRP-conjugated anti-S tag antibody. (C) MMP-9 inhibitory activity of M91005 was measured by enzymatic assay using peptide substrate. Active MMP-9_Cat-H6 (0.4 nM) was incubated with various concentrations of M91005 (0–25 nM) for 1 h at 37°C, and then peptide substrate 3226-v (10 μM) was added. MMP-9 activity was determined by monitoring the hydrolysis of the peptide substrate, and the activity in the absence of M91005 was taken as 100%. (D) MMP-9 inhibitory activity of engineered SPINK2-derived inhibitors was measured by enzymatic assay using the macromolecular substrate. Active MMP-9_Cat-H6 (0.6 nM) was incubated with various concentrations of inhibitors (0–100 nM) for 1 h at 37°C, and then DQ-gelatin (10 μg/ml) was added. MMP-9 activity was determined by monitoring the hydrolysis of DQ-gelatin and the activity in the absence of inhibitor was taken as 100%. Data are shown as the mean of duplicate experiments in (A) and the mean ± S.D. (n = 3) in (B–D). All curves were obtained by non-linear curve fitting as described in “Materials and methods”.

We evaluated the binding and inhibitory activity of each inhibitor. In ELISA, all inhibitors showed strong binding activities to active MMP-9 in a concentration-dependent manner (Fig 1A) and their EC_50_ values were double-digit nM (Table 1). These inhibitors did not show any binding to pro-MMP-9 at concentrations up to 1 μM (Fig 1B), while anti-MMP-9 antibody GS-5745 [37] bound to both active and pro-MMP-9 (S3 Fig). In the enzyme assay using the peptide substrate, all inhibitors strongly inhibited MMP-9 activity and the *K*_*i*_ values of all inhibitors were low single-digit nM (Fig 1C and Table 1). They also showed MMP-9 inhibitory activity in the enzyme assay using gelatin as a protein substrate (Fig 1D and Table 1). These results demonstrated that the obtained MMP-9 inhibitors had potent inhibitory activity.

**Table 1:** Binding affinity and inhibitory activity of inhibitors against active MMP-9.

Clone	ELISA EC_50_ (nM)^a^	Peptide	Gelatin	
		*K*_*i*_ (nM)^b^	IC_50_ (nM)^c^	R^2^
M91002	47	2.3 ± 0.6	8.5 ± 2.1	0.984
M91005	46	1.4 ± 0.2	8.2 ± 1.2	0.991
M91011	16	1.6 ± 0.1	5.0 ± 0.5	0.993
M91012	46	2.1 ± 0.1	8.6 ± 1.7	0.993
sc-311438	N/A^d^	1.5 ± 0.2	2.3 ± 0.1	0.997

^*a*^EC_50_ was determined by ELISA as the mean (n = 2).

^*b*^*K*_*i*_ was determined by the MMP-9 inhibitory assay with 10 μM peptide substrate 3226-v. The *K*_*i*_ values are shown as the mean ± standard deviation (S.D.) (n = 3).

^*c*^IC_50_ was determined by the MMP-9 inhibitory assay with 10 μg/ml DQ-gelatin substrate. The IC_50_ values are shown as the mean ± S.D. (n = 3).

^*d*^N/A denotes “not applicable”.

**Fig 2:**
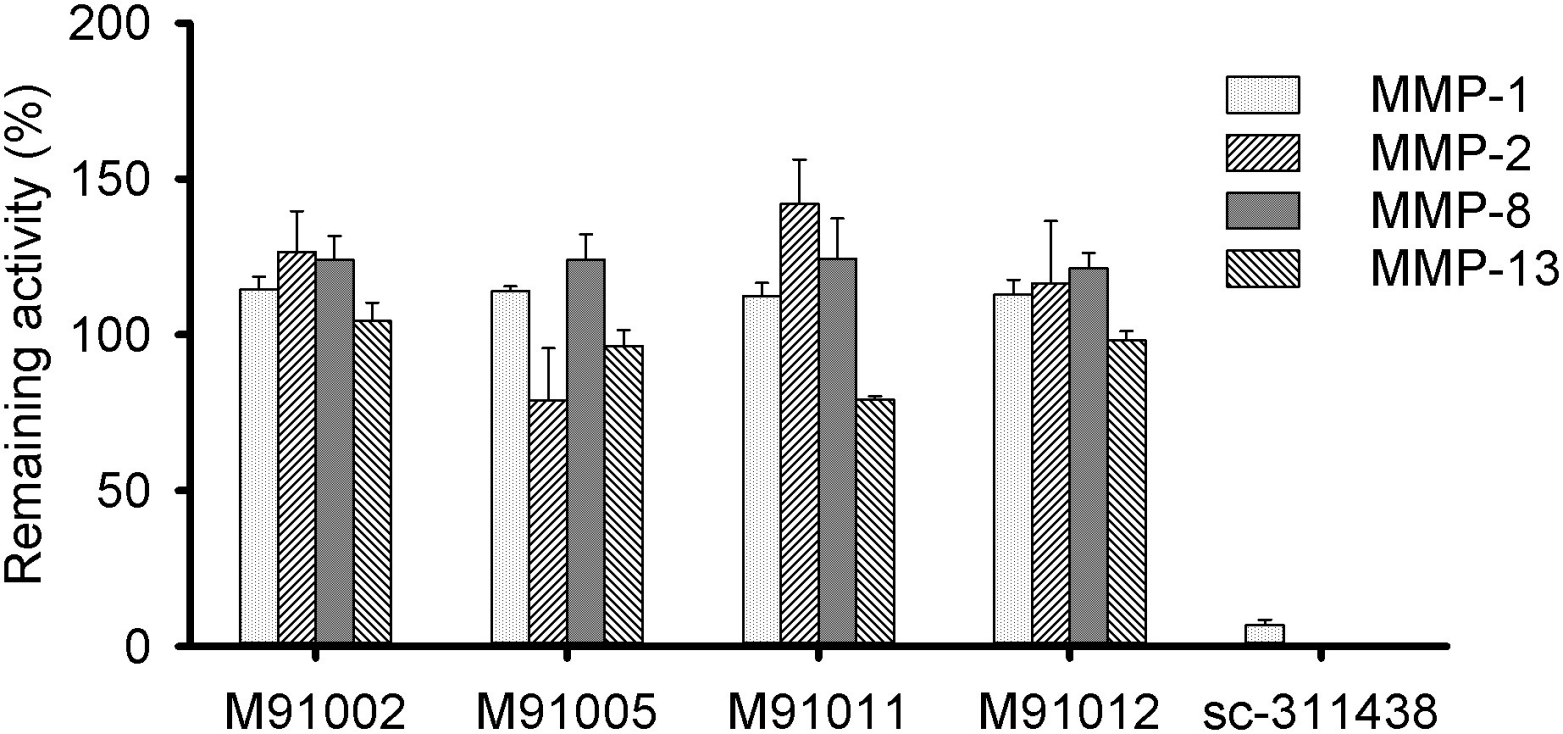
Cross-reactivity of engineered SPINK2-derived inhibitors against MMP-9. The cross-reactivities against MMP-1, -2, -8, and -13 were measured by enzymatic assay using the peptide substrate. Each active MMP (MMP-1, 5 nM; MMP-2, 1.4 nM; MMP-8, 0.6 nM; MMP-13, 2 nM) was incubated with inhibitors (1 μM) for 10 min (for MMP-13) or 60 min (for MMP-1, -2, and -8) at 37°C, and then peptide substrate 3226-v (10 μM for MMP-1, -8, and -13, or 50 μM for MMP-2) was added. Enzymatic activity was determined by monitoring the hydrolysis of the peptide substrate, and each remaining enzymatic activity was normalized to the activity in the absence of inhibitors. Each *bar* represents the mean ± S.D. (n = 3).

For the cross-reactivity test, we evaluated the inhibitory activities against MMP-1, -2, -8, and -13, the primary sequence in the catalytic domain of each of which is similar to that of MMP-9 (S4 Fig). Although the chemical active-site inhibitor sc-311438 completely inhibited all of the MMPs, our MMP-9 inhibitors did not inhibit them at a final concentration of 1 μM (Fig 2). Additionally, they did not show any inhibitory activity against the other tested MMPs (S1 Table). Therefore, the obtained MMP-9 inhibitors showed potent inhibition with high specificity to MMP-9.

### M91005 interacts with catalytic domain of MMP-9

The question arises as to why, despite MMPs showing high sequence homology with each other, the obtained inhibitors achieved high specificity. To address this, we investigated how the inhibitor recognizes MMP-9 using the most potent inhibitor, M91005.

At the beginning of this investigation, we examined which domain of MMP-9 was involved in the interaction with M91005. MMP-9 inhibitory assays were performed using full-length MMP-9, the form with the HPX domain deleted (MMP-9_Cat), and the form with both Fn-like domain and HPX domain deleted (the catalytic domain). M91005 inhibited all forms of active MMP-9 with similar inhibitory activities (S7 Fig), suggesting that it interacts with the catalytic domain but not the Fn-like domain or the HPX domain. Therefore, we focused on the catalytic domain in the following analysis.

### M91005 binds to catalytic glutamate residue and active-site cleft of MMP-9

We investigated which region of the catalytic domain contributes to the interaction with M91005. Given that M91005 bound not to pro-MMP-9 but to active MMP-9, it would be expected to bind to the region including the active site and the active-site cleft, which are hidden by the pro-peptide in pro-MMP-9.

**Fig 3:**
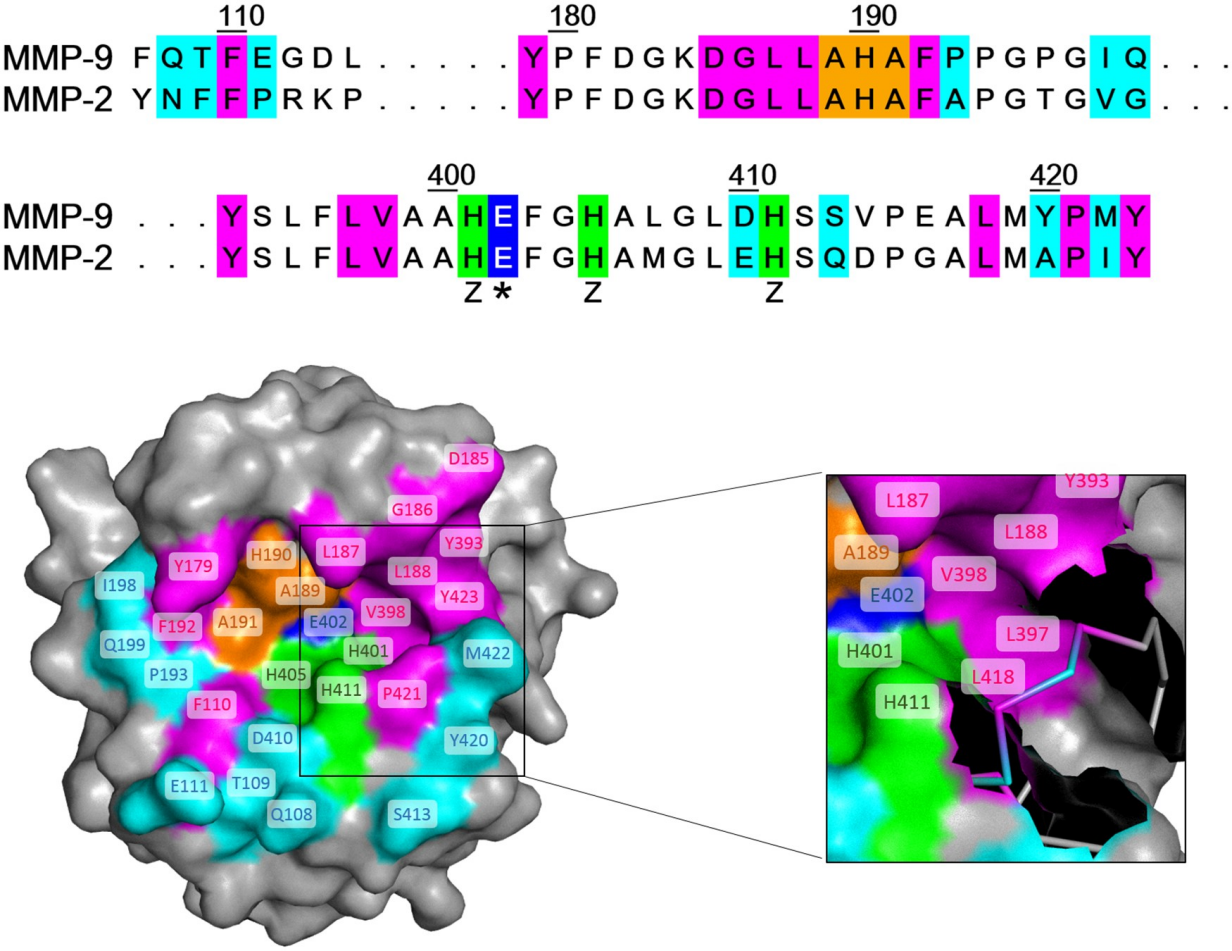
Design of MMP-9 mutants for analysis of interaction between MMP-9 and M91005. (Top) The sequence alignment between MMP-9 and MMP-2 in the active-site cleft and exosite of MMP-9. The residues are numbered according to the generic MMP-9 nomenclature. Catalytic Glu-402 is indicated by an *asterisk* and shown in *blue*. Three histidine residues (His-401, His-405, and His-411) that chelate catalytic zinc ion are indicated by a “Z” and shown with a *green* background. Alanine substitution sites in the active-site cleft (for cleft mutants) and MMP-9/-2 chimeric mutation sites in the exosite (for exosite mutants) are highlighted in *magenta* and *cyan*, respectively. Three residues (Ala-189, His-190, and Ala-191) in the active-site cleft, the side chain of which is not exposed to solvent, are shown with an *orange* background. (Bottom) The structure of the catalytic domain of active MMP-9, colored in *gray* (Protein Data Bank code 4H3X). Each residue is shown with *coloring* as in the *top panel*.

**Fig 4:**
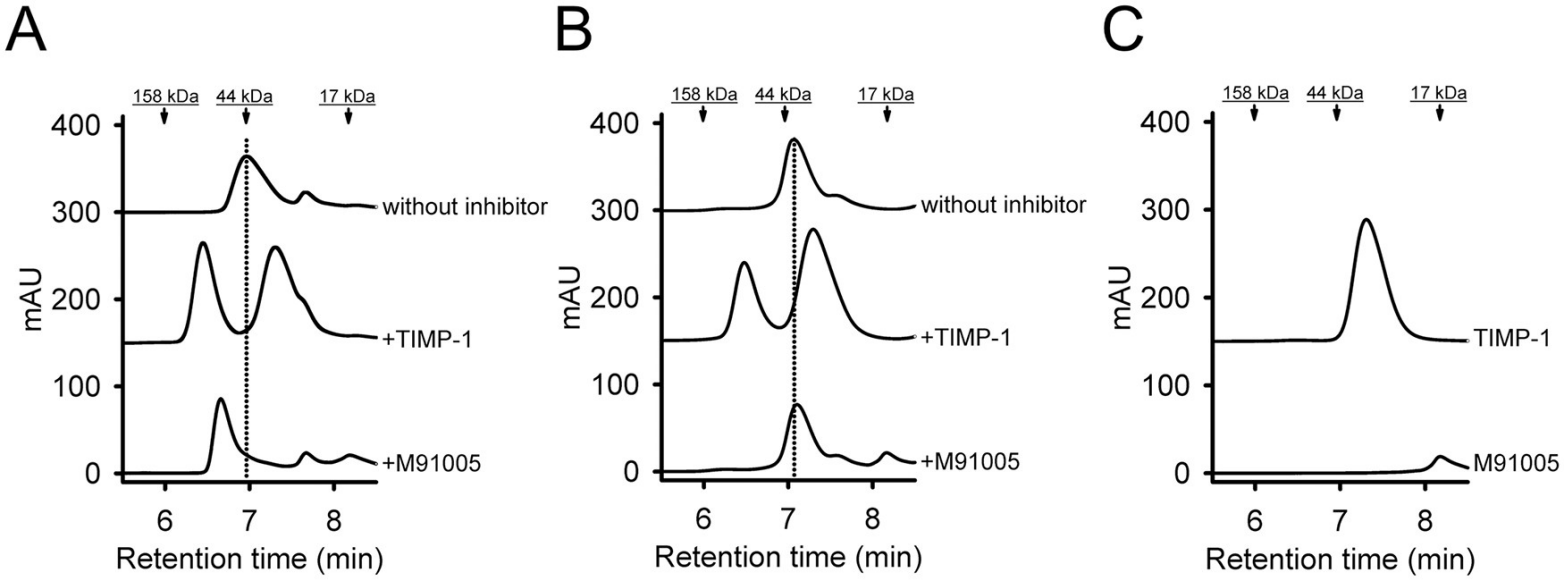
M91005 binds to the active site of MMP-9. The binding activity to the catalytic Glu-402 residue of MMP-9 was evaluated by SEC. Active MMP-9_Cat-H6 or MMP-9_Cat-E402Q-H6 (25 μM each) was incubated with TIMP-1 or M91005 (75 μM each) in PBS for 1 h at 4°C, and then 10 μl of the reaction mixture was analyzed by monitoring the absorbance at 280 nm in SEC. Chromatograms are shown in panels as follows: active MMP-9_Cat-H6 (A), MMP-9_Cat_E402Q-H6 (B), and no MMP-9 (C). *Dotted lines* indicate the retention time (RT) of active MMP-9_Cat-H6 (7.0 min, A) and MMP-9_Cat_E402Q-H6 (7.1 min, B). Molecular weights (158, 44, and 17 kDa) of the gel filtration standard are indicated at the top of each panel. The RT of active MMP-9_Cat-H6 but not MMP-9_Cat-E402Q-H6 was shifted to a high molecular weight by incubation with M91005. *mAU*, milliabsorbance units.

First, to reveal whether M91005 recognizes the active site, we performed the assay using the active-site mutant of MMP-9. The active site of MMP-9 consists of a catalytic zinc ion, catalytic glutamate residue (Glu-402; Fig 3 *blue*), and three histidine residues (His-401, His-405, and His-411; Fig 3 *green*) chelating catalytic zinc. Four kinds of active-site mutants (H401A, E402Q, H405A, and H411A) were constructed in accordance with a previous report [38]. Pro-MMP-9_Cat_E402Q-H6 was well expressed in HEK293F cells, while the other active-site mutants H401A, H405A, and H411A were not expressed (S8 Fig). Similar to the wild-type MMP-9 (pro-MMP-9_Cat-H6), pro-MMP-9_Cat_E402Q-H6 was successfully processed by MMP-3 (S1B Fig). Because the pro-peptide-removed E402Q mutant (MMP-9_Cat_E402Q-H6) does not show any proteolytic activity [39], the interaction between M91005 and Glu-402 was confirmed by not the enzymatic assay but the SEC binding assay. By pre-incubation with TIMP-1 or M91005, the retention time of the peak of active MMP-9_Cat-H6 was shifted from 7.0 min to 6.4 or 6.7 min, respectively (Fig 4A). These findings showed that TIMP-1 and M91005 bound to active MMP-9_Cat-H6. In the case of MMP-9_Cat_E402Q-H6, the retention time of the peak was shifted from 7.1 min to 6.5 min by pre-incubation with TIMP-1, but not shifted by pre-incubation with M91005 (Fig 4B). M91005 interacted with active MMP-9_Cat-H6 but not with MMP-9_Cat_E402Q-H6, suggesting that M91005 directly recognized the catalytic Glu-402 residue of MMP-9.

**Fig 5:**
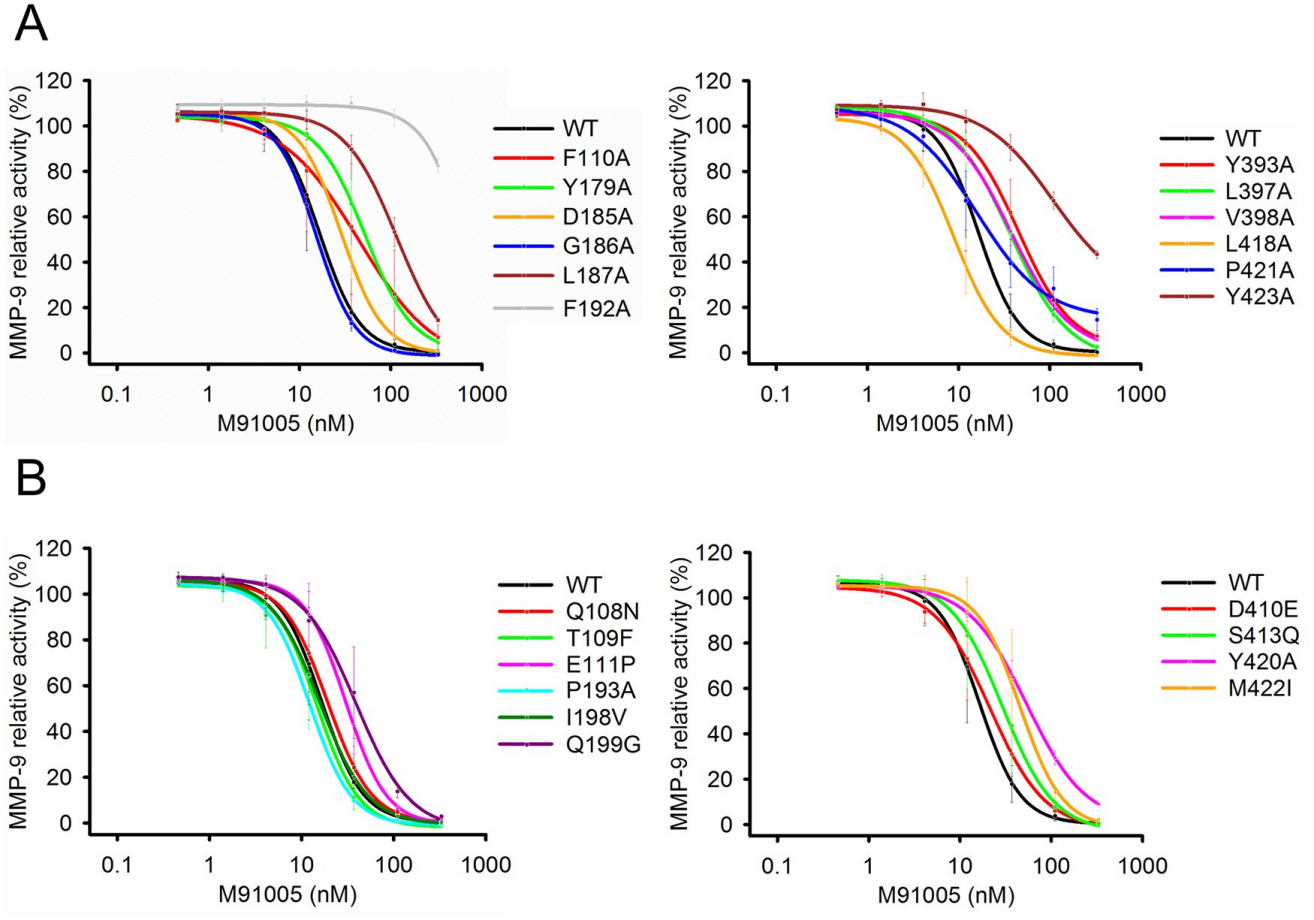
M91005 recognizes residues in the active-site cleft and the exosite of MMP-9. MMP-9 inhibitory activities of M91005 towards the cleft mutants (A) and the exosite mutants (B) were measured by enzymatic assay using peptide substrate. Active MMP-9_Cat (WT) or the MMP-9 mutants activated by EK (1 nM each) were incubated with various concentrations of M91005 (0–330 nM or 0–1,000 nM) for 1 h at 37°C. Enzymatic activities were determined by monitoring the degradation of peptide substrate 3226-v (10 μM). Each enzymatic activity was normalized to the activity of the MMP-9 mutant without M91005. Data are shown as the mean ± S.D. (n = 3). All curves were obtained by non-linear curve fitting.

Next, to explore the interacting residues in the region surrounding the active site, the so-called active-site cleft, we designed MMP-9 mutants with mutations in the active-site cleft and carried out enzymatic assays using them. To obtain a comprehensive understanding of the interacting residues, we selected 13 residues (Phe-110, Tyr-179, Asp-185, Gly-186, Leu-187, Leu-188, Phe-192, Tyr-393, Leu-397, Val-398, Leu-418, Pro-421, and Tyr-423; Fig 3 *magenta*) that met the following criteria: (i) involved in interaction with peptide substrate based on the structural information of the complex of MMP-9 and peptide substrate reported previously (Protein Data Bank code 4JIJ), (ii) identical to those of MMP-2, and (iii) having a side chain exposed to the solvent. These residues were mutated to alanine as the cleft mutants. The N-terminal residue of the cleft mutants was designed to be Phe to mimic the N-terminus after digestion by MMP-3 [6]. For convenient preparation of these mutants, an enterokinase (EK) recognition site was inserted between the pro-peptide and the N-terminus of the catalytic domain (Phe-107), resulting in the mutants being uniformly processed and highly purified (S5A and S5B Fig). Except for the L188A mutant, all of the cleft mutants with sufficient proteolytic activity could be prepared (S5C Fig). In enzymatic assays using the peptide substrate, F110A, Y179A, L187A, F192A, Y393A, and Y423A mutants significantly weakened the inhibitory activities of M91005 compared with that of wild-type MMP-9 (Fig 5A and Table 2). These results suggested that M91005 also interacted with Phe-110, Tyr-179, Leu-187, Phe-192, Tyr-393, and Tyr-423 residues, located in the active-site cleft of MMP-9.

**Table 2:** Inhibitory activity of M91005 against cleft mutants.

MMP-9	IC_50_ (nM)^a^	R^2^	*P* value^b^
WT	17 ± 7	0.965	-
F110A	69 ± 12	0.845	<0.001
Y179A	55 ± 13	0.989	<0.001
D185A	29 ± 5	0.990	0.69
G186A	15 ± 0	0.999	0.9997
L187A	120 ± 10	0.991	<0.001
F192A	˃ 1,000	0.926	<0.001
Y393A	48 ± 14	0.985	0.0085
L397A	37 ± 10	0.986	0.15
V398A	39 ± 3	0.999	0.10
L418A	9.3 ± 2.9	0.988	0.96
P421A	23 ± 9	0.961	0.99
Y423A	230 ± 20	0.980	<0.001

^*a*^IC_50_ was determined by the MMP-9 inhibitory assay with 10 μM peptide substrate 3226-v. The IC_50_ values are shown as the mean ± S.D. (n = 3).

^*b*^Statistical analysis of cleft mutants *versus* WT was performed by one-way ANOVA with Dunnett’s post tests for multiple comparisons.

### M91005 recognizes the exosite in the catalytic domain of MMP-9

**Fig 6:**
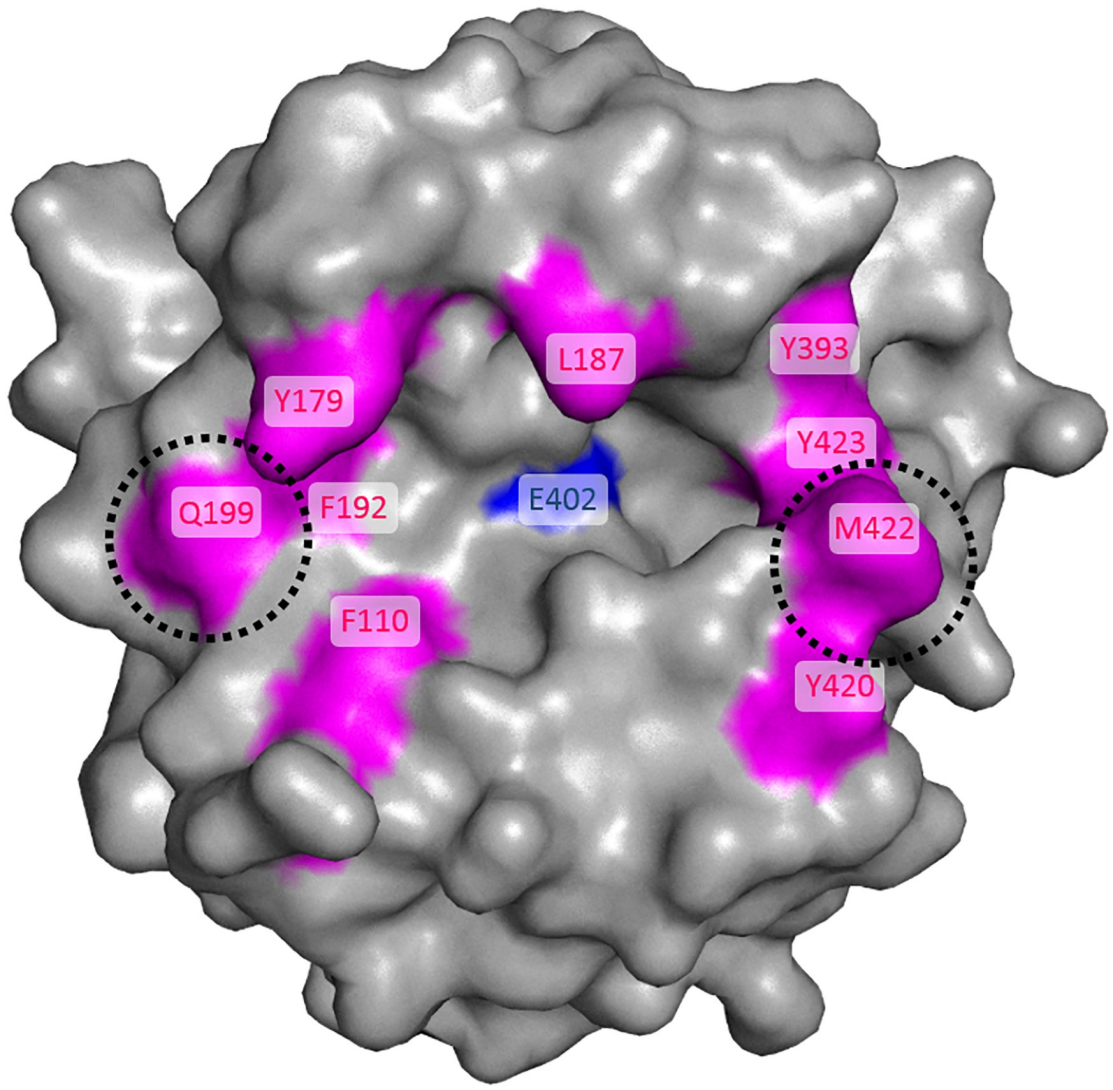
Mapping of the possible interaction sites with M91005 onto the structure of active MMP-9. In accordance with the results of binding and enzymatic assay using MMP-9 mutants, the residues identified as being involved in the interaction with M91005 were mapped onto the structure of the catalytic domain of active MMP-9 (*gray*, Protein Data Bank code 4H3X). The results of the binding assays (Fig 4) suggested that catalytic Glu-402 (*blue*) contributed to the interaction between M91005 and MMP-9. The results of enzyme inhibitory assays (Fig 5, Tables 2 and 3) suggested that M91005 recognized Phe-110, Tyr-179, Leu-187, Phe-192, Gln-199, Tyr-393, Tyr-420, Met-422, and Tyr-423 (*magenta*). The interaction with characteristic residues, Gln-199 and Met-422 (*black dotted circle*), is likely to contribute to the specific inhibition of M91005 toward MMP-9.

Considering the high specificity of M91005 despite the high homology in amino acid sequences and structures of MMPs, M91005 should interact not only with the active site and the active-site cleft but also with an additional region in the catalytic domain that differentiates MMP-9 from the other MMPs. M91005 inhibited MMP-9 but not MMP-2, suggesting that M91005 recognizes structural differences between these two members of the MMP family in the form of an exosite. To investigate this hypothesis, we designed MMP-9/-2 chimeric mutants as the exosite mutants. Ten residues (Gln-108, Thr-109, Glu-111, Pro-193, Ile-198, Gln-199, Asp-410, Ser-413, Tyr-420, and Met-422; Fig 3 *cyan*) were selected based on the following criteria: (i) being near the active-site cleft and (ii) not being identical to that of MMP-2. These residues were mutated to the corresponding amino acid of MMP-2. All exosite mutants were well expressed in HEK293F cells, and then purified pro-form proteins were efficiently activated by using EK by the same method as for the cleft mutants (S5 Fig). In enzymatic assays using the peptide substrate, Q199G, Y420A, and M422I mutants significantly weakened the inhibitory activities of M91005 compared with that of wild-type MMP-9 (Fig 5B and Table 3). These results suggested that M91005 also interacted with Gln-199, Tyr-420, and Met-422, which were located away from the active site of MMP-9. Collectively, catalytic Glu-402 (Fig 6 *blue*), Phe-110, Tyr-179, Leu-187, Phe-192, Gln-199, Tyr-393, Tyr-420, Met-422, and Tyr-423 (Fig 6 *magenta*) were suggested to be interacted with M91005.

**Table 3:** Inhibitory activity of M91005 against exosite mutants.

MMP-9	IC_50_ (nM)^a^	R^2^	*P* value^b^
WT	17 ± 7	0.965	-
Q108N	20 ± 6	0.981	0.9995
T109F	15 ± 4	0.977	0.9995
E111P	31 ± 9	0.983	0.50
P193A	13 ± 3	0.992	0.999
I198V	16 ± 4	0.989	0.9998
Q199G	40 ± 16	0.967	0.043
D410E	22 ± 9	0.975	0.999
S413Q	30 ± 14	0.949	0.50
Y420A	54 ± 8	0.995	<0.001
M422I	45 ± 18	0.959	<0.001

^*a*^IC_50_ was determined by the MMP-9 inhibitory assay with 10 μM peptide substrate 3226-v. The IC_50_ values are shown as the mean ± S.D. (n = 3).

^*b*^Statistical analysis of exosite mutants *versus* WT was performed by one-way ANOVA with Dunnett’s post tests for multiple comparisons.

## Discussion

In this study, we succeeded in obtaining MMP-9 inhibitors with high potency and specificity from our proprietary engineered SPINK2 phage display library without any affinity maturation. The potency and specificity of M91005 were comparable to those of the MMP-9 inhibitory antibody CALY-001 [40]. CALY-001 did not bind to pro-MMP-9; however, it showed non-competitive inhibition against MMP-9, suggesting that it is not an active-site inhibitor. On the other hand, M91005 did not interact with the active-site mutant MMP-9_Cat_E402Q (Fig 4B), suggesting that it may recognize the active site. The result that M91005 bound to active MMP-9 and not to pro-MMP-9 (Fig 1B) supports the view that M91005 recognizes the active site. Taking these findings together, the recognition mechanism of M91005 differs from that of CALY-001. To our knowledge, M91005 is the first MMP-9-specific inhibitor, based on protein, to recognize the active site.

It was strongly suggested that M91005 interacted with the active site; however, it remained unclear why M91005 achieved specific inhibition toward MMP-9 despite the active sites being highly conserved among MMP family members. To understand the mechanism behind this high specificity, we analyzed the binding sites by assays using several MMP-9 mutants having alanine substitution in the active-site cleft (cleft mutants) and MMP-9/-2 chimeric mutation in the exosite (exosite mutants). For the enzymatic assay using MMP-9 mutants, it is critically important that they have sufficient purity and enzymatic activity. To create activated MMP-9 mutants with high stability, we found that autolysis of activated MMP-9, which occurred upon long-term incubation at 25°C (S10 Fig), was prevented by activation using EK at 4°C. In most of the mutants, despite their good purity and processing by EK, the cleavage activities of peptide substrates were reduced compared with that of the wild-type (S5C Fig); however, in our preliminary experiment, V398A, L418A, and P421A mutants significantly affected the inhibitory activities of sc-311438 compared with that of wild-type MMP-9 (S6A Fig), consistent with the previous report describing that sc-311438 interacts with the S1′ pocket of MMPs [41], which was formed by residues including Val-398, Leu-418, and Pro-421 in MMP-9. Therefore, we concluded that enzymatic assays using these mutants are available to explore the residues of MMP-9 interacting with the inhibitors.

The results using the cleft mutants (Fig 5A and Table 2) strongly suggested that M91005 recognized Phe-192 and Tyr-423. As they are conserved in MMP-1, -2, -8, -9, and -13 (S4 Fig), additional recognition sites are required to achieve the specific inhibition of MMP-9. For further analysis, we selected MMP-2, which has the highest homology to MMP-9 among MMP family proteins, and performed enzymatic assays using exosite mutants. The results (Fig 5B and Table 3) suggested that M91005 recognized Gln-199, Tyr-420, and Met-422, which are characteristic residues of MMP-9 compared with the other MMPs. In terms of their location, Gln-199 is near Phe-192 and Met-422 is near Tyr-423 (Fig 6), consistent with the suggestion that M91005 recognizes both Phe-192 and Tyr-423. Interestingly, both Gln-199 and Met-422 are unique sequences that are found only in MMP-9 among the five MMP family proteins (S4 Fig). It was suggested that M91005 recognizes not only highly homologous Phe-192 and Tyr-423 but also unique Gln-199 and Met-422 located near these residues. Therefore, it was in turn suggested that M91005 specifically inhibits MMP-9 by recognizing both Gln-199 and Met-422 in the exosite (Fig 6 *black dotted circle*).

Gln-199 and Met-422, which are distant from each other, are located on the non-prime side and prime side of the catalytic domain of MMP-9, respectively. The side chain of Gln-199 is thought to be involved in forming the S4 subsite of MMP-9 [42,43]. Met-422 forms a groove recognizing the region from the P2′ to the P4′ position of the substrate [44]. Thus, it was suggested that M91005 interacts with the region including S4 and the S4′ subsite as well as the active site of MMP-9. Based on the structural information of MMP-9, the distance from Cα of Gln-199 to that of Met-422 is over 20 Å. This is a long distance comparable to the large interface between KLK4 and the engineered SPINK2-derived KLK4 inhibitor, K41043, which we previously demonstrated [24]. K41043 recognized the large surface area containing both the S1 pocket of KLK4 and the region surrounding the pocket, and showed high specificity. Similar to K41043, the large interaction area generated by the randomized loop presented by the engineered SPINK2 may have allowed M91005 to recognize both active site and exosite of MMP-9. Nam *et al*. reported that the engineered antibody Fab 3A2, which accessed the active site of target MMP-14 and showed high specificity, recognized the Phe-260 residue, which forms the S1′ subsite of MMP-14 [45]. Phe-260 of MMP-14 corresponds to Met-422 of MMP-9 in the sequence alignment and is a characteristic residue in the exosite of MMP-14. The importance of exosite recognition for high specificity has also been suggested in another report, supporting our finding that the binding of M91005 to the exosite contributed to its specificity to MMP-9 in this study.

We speculated that M91005 interacted with Gln-199 and Met-422 of MMP-9 by enzymatic assays using the MMP-9 mutants. ELISA or enzymatic assays using mutant enzymes provided some insights into the interacting residues of the inhibitors in previous reports [45–48]. Similarly, our results using the MMP-9 mutants contributed to providing meaningful information regarding the mechanism by which M91005 recognizes MMP-9 in this research. Future investigation including X-ray crystallographic analysis of the complex of M91005 and MMP-9 may offer additional insights into the mechanism behind the potent and specific inhibition of MMP-9 by M91005.

Using the engineered SPINK2 library, we obtained MMP-9-specific inhibitors with high potency, and they recognized exosites distant from each other in addition to the active site, which could be achieved by the large interaction area provided by the loop of the SPINK2 scaffold. The characteristic recognition mechanism shown by engineered SPINK2-derived inhibitors may also be useful in the creation of specific active-site inhibitors of other MMPs, and the engineered SPINK2 library may contribute to the discovery of specific inhibitors useful for basic and clinical research on MMPs.

## Materials and methods

### Reagents

Fluorescence-quenching peptide substrates, MOCAc-Arg-Pro-Lys-Pro-Val-Glu-Nva-Trp-Arg-Lys(Dnp)-NH_2_ (3168-v, Lot No. 610613) and MOCAc-Lys-Pro-Leu-Gly-Leu-A_2_pr(Dnp)-Ala-Arg-NH_2_ (3226-v, Lot No. 650414), were purchased from Peptide Institute. Dye-quenched (DQ) gelatin (D-12054, Lot No. 1694715) was obtained from Thermo Fisher Scientific. The broad MMP inhibitor *N*-hydroxy-1-(4-methoxyphenyl)sulfonyl-4-(4-biphenylcarbonyl)piperazine-2-carboxamide (sc-311438) was obtained from Santa Cruz Biotechnology.

The full-length active hMMP-2 and active hMMP-15 catalytic domain were purchased from EMD Millipore. The catalytic domains of active hMMP-7, active hMMP-10, and active hMMP-12 were obtained from Enzo Life Sciences. Active hMMP-14 catalytic domain was purchased from Thermo Fisher Scientific. The catalytic domains of pro-hMMP-16 and pro-hMMP-17 were purchased from R&D Systems. Pro-MMP-16 was activated by Furin, and pro-MMP-17 was activated by 4-aminophenylmercuric acetate (APMA; Sigma-Aldrich), in accordance with each manufacturer’s protocol.

### Protein expression and purification

All pro-MMPs with the native leader sequences were cloned into pcDNA3.3 vector (Thermo Fisher Scientific) and their constructs were as follows. Each catalytic domain of pro-hMMP-1 (residues 20–261), pro-hMMP-8 (residues 21–262), or pro-hMMP-13 (residues 20–267) was fused to the N-terminal His_6_ tag. Pro-hMMP-9 catalytic domain with an insertion of fibronectin-like domain (residues 20–449; pro-MMP-9_Cat) was fused to the C-terminal His_6_ tag (pro-MMP-9_Cat-H6). For preparation of biotinylated MMP-9 protein, pro-MMP-9_Cat was fused to the N-terminal His_6_ tag, C-terminal FLAG tag, and biotin acceptor peptide (pro-MMP-9_Cat-BAP). For activation of pro-MMP-9 using enterokinase (EK), pro-MMP-9_Cat was fused to the N-terminal His_6_ tag, followed by insertion of the EK cleavage site (amino acid sequence: DDDDK) between Arg-106 and Phe-107 (pro-EK-MMP-9_Cat). Pro-MMP-9_Cat cleft mutants (pro-EK-MMP-9_Cat_F110A, Y179A, D185A, G186A, L187A, L188A, F192A, Y393A, L397A, V398A, L418A, P421A, and Y423A) and pro-MMP-9/MMP-2 chimeric mutants (pro-EK-MMP-9_Cat_Q108N, T109F, E111P, P193A, I198V, Q199G, D410E, S413Q, Y420A, and M422I) were constructed from pro-EK-MMP-9_Cat by single-amino-acid substitution using KOD -Plus- Mutagenesis Kit (TOYOBO). Pro-MMP-9 E402Q mutant (pro-MMP-9_Cat_E402Q-H6) was generated from pro-MMP-9_Cat-H6 using QuikChange Site-Directed Mutagenesis Kit (Agilent).

All pro-MMPs were transiently expressed in HEK293F cells (Thermo Fisher Scientific). Six days after transfection, all pro-MMP proteins were purified using HisTrap excel gel (GE Healthcare), followed by buffer exchange into PBS. Pro-EK-MMP-9_Cat and mutants were further purified using gelatin-Sepharose resin (GE Healthcare), followed by buffer exchange into PBS. Pro-MMP-9_Cat-BAP was biotinylated using biotin-protein ligase BirA (Avidity), in accordance with the manufacturer’s protocol.

Human TIMP-1 (residues 1–207) with a C-terminal FLAG tag was cloned into the pcDNA3.3 vector. TIMP-1 was transiently expressed in HEK293F cells and purified using ANTI-FLAG M2 Affinity Gel (Sigma-Aldrich), followed by buffer exchange into PBS.

### Activation of MMP

MMP-1, -3, -8, and -13 were activated using APMA in TNC buffer (50 mM Tris-HCl, 200 mM NaCl, 2 mM CaCl_2_, pH 7.5) at 37°C as follows. Active forms of MMP-1 and MMP-8 were generated by incubation of pro-MMP-1 (15.8 μM) or pro-MMP-8 (15.9 μM) with 500 μM APMA for 1 h. Active MMP-3 was generated by incubation of pro-MMP-3 (9.4 μM) with 500 μM APMA for 4 h. Active MMP-13 was generated by incubation of pro-MMP-13 (15.5 μM) with 50 μM APMA for 1 h. After activation of all MMPs using APMA, buffers were exchanged into PBS at 4°C using Sephadex G-25 (GE Healthcare).

For MMP-9 activation using MMP-3, the final 10.2 μM pro-MMP-9_Cat-H6 or biotinylated pro-MMP-9_Cat-BAP was incubated with 1.1 μM active MMP-3 in TNC buffer for 4 h at 37°C. For MMP-9 activation using EK, the final 4 μM pro-EK-MMP-9_Cat or mutants were incubated with 32 U/ml EKMax Enterokinase (Thermo Fisher Scientific) in the cleavage buffer (20 mM Tris-HCl, 50 mM NaCl, 2 mM CaCl_2_, pH 7.4) for 2 h at 4°C. EK was removed by EKapture Agarose (EMD Millipore). After the activation of MMP-9, buffers were exchanged into PBS at 4°C using an Amicon-Ultra centrifugal filter (10,000 NMWL; EMD Millipore). The purity of each active MMP-9 mutant was confirmed by SDS-PAGE.

For the preparation of MMP-9 E402Q mutant (MMP-9_Cat_E402Q-H6) the pro-peptide of which was removed, a final concentration of 10.2 μM pro-MMP-9_Cat_E402Q-H6 was incubated with 3.4 μM active MMP-3 in TNC buffer for 4 h at 37°C. After incubation, buffer was exchanged into PBS at 4°C using Amicon-Ultra centrifugal filter (10,000 NMWL). The purity of MMP-9_Cat_E402Q-H6 was confirmed by SDS-PAGE.

### Phage panning

The engineered SPINK2 phage library (functional diversity, 1.2 × 10^10^) was previously established, and phage panning was performed following methods described previously [24]. Briefly, for the first round of panning, 50 nM biotinylated active MMP-9_Cat-BAP was immobilized on Dynabeads M-280 Streptavidin (Thermo Fisher Scientific) for 2 h at 4°C. The beads were blocked with 3% BSA in TNC-T buffer (0.05% Tween 20 in TNC buffer) for 1 h at 4°C. Then, the engineered SPINK2 phage library (approximately 1.8 × 10^13^ phages) was incubated with active MMP-9_Cat-BAP immobilized on beads in 3% BSA in TNC-T buffer overnight at 4°C. The beads were washed with TNC-T buffer, and then the bound phages were eluted using AcTEV Protease (Thermo Fisher Scientific). The recovered phage repertoire was amplified in *Escherichia coli* XL1-Blue cells (Agilent Technologies), which were then subjected to the next round of panning. In the second and third rounds, the amplified phages were incubated with 5 nM biotinylated active MMP-9_Cat-BAP at 4°C overnight. Phages bound to biotinylated active MMP-9_Cat-BAP were collected using Dynabeads M-280 Streptavidin blocked with 3% BSA in TNC-T buffer for 30 min at 4°C. Bound phages were washed with TNC-T buffer, followed by elution using AcTEV Protease. During subsequent selection rounds, the number of washing steps was gradually increased.

### Expression and purification of inhibitors

Engineered SPINK2-derived MMP-9 inhibitors were produced following methods described previously [24]. Briefly, the enriched phagemid DNA was cloned into the EcoRI/NotI sites of the expression vector pET32a (EMD Millipore). Origami B (DE3) (EMD Millipore) was transformed with the resulting vectors. The transformed *E*. *coli* was cultured in 2-YT medium (Thermo Fisher Scientific) at 37°C; then, isopropyl β-D-1-thiogalactopyranoside (IPTG) was added to a final concentration of 1 mM for induction. After cultivation at 16°C overnight, the cell pellet was collected and lysed using BugBuster Master Mix (EMD Millipore). His_6_ tag-fused inhibitor with an N-terminal thioredoxin tag was purified using TALON Metal Affinity Resin (Clontech). The purified proteins were used for the primary screening of inhibitors. For further evaluation of the inhibitors, thioredoxin and His_6_ tag were removed using Thrombin Cleavage Capture Kit (EMD Millipore). The inhibitors were finally purified by affinity chromatography using TALON Metal Affinity Resin, followed by size-exclusion chromatography (SEC) using Superdex 75 (GE Healthcare). The purified inhibitors had an N-terminal S tag for detection.

### ELISA for MMP-9 binding of inhibitors

Biotinylated pro-MMP-9_Cat-BAP or biotinylated active MMP-9_Cat-BAP (each 50 nM) in PBS was coated on a 96-well Nunc Immobilizer streptavidin clear plate (Thermo Fisher Scientific) for 2 h at 4°C. The plate was washed with PBS-T (0.05% Tween 20 in PBS) and blocked with 3% BSA in PBS-T for 1 h at room temperature. Then, twofold serially diluted inhibitors (1–1,000 nM) in PBS-T were added and incubated for 90 min at room temperature. After washing with PBS-T, horseradish peroxidase (HRP)-conjugated anti-S tag antibody (Bethyl Laboratories) was added (1:10,000 dilution in PBS-T) and incubated for 1 h at room temperature. After washing with PBS-T, the reaction was developed with 2,2′-azino-di-(3-ethylbenzthiazoline sulfonic acid) substrate (Nacalai Tesque) at room temperature. The absorbance at 405 nm was measured using an EnSpire fluorescence plate reader (PerkinElmer). The EC_50_ values were determined by a four-parameter logistic fit of the absorbance data using GraphPad Prism version 5.0 (GraphPad Software). Results are means of at two independent experiments.

### Size-exclusion chromatography analysis for MMP-9 binding of inhibitors

The purified inhibitor and active MMP-9_Cat-H6 or MMP-9_Cat_E402Q-H6 were incubated at a molar ratio of 3:1 for 1 h at 4°C. Then, they were analyzed by SEC using an ACQUITY UPLC BEH200 column (Waters) in PBS. Gel filtration standard (Bio-Rad) was used as a molecular weight marker.

### Enzymatic assays

All enzymatic assays were carried out at 37°C in a 96-well PROTEOSAVE black plate (Sumitomo Bakelite). As the assay buffer, TC buffer (50 mM Tris-HCl, 10 mM CaCl_2_, pH 7.5) was used in the MMP-17 enzymatic assay, while TNC buffer was used in the enzymatic assays for the other MMPs. The increase of fluorescence signal of the substrate was measured on EnSpire. The excitation and emission wavelengths of each substrate were as follows: 328 and 393 nm for 3168-v and 3226-v; and 495 and 515 nm for DQ-gelatin. Initial reaction velocities were determined by a linear fit of raw experimental data traces (fluorescence *versus* time) as the slopes of the regression lines. The IC_50_ values were calculated from a four-parameter logistic fit of the initial reaction velocities using GraphPad Prism.

For the primary screening of inhibitors, each clone was incubated with 0.6 nM active MMP-9_Cat-H6 for 10 min. Then, peptide substrate 3226-v was added to a final concentration of 10 μM. The IC_50_ values were calculated from at least two independent experiments.

For the enzymatic assay with MMP-9 mutants activated by EK, 1 nM active MMP-9_Cat or mutants were incubated with threefold serially diluted inhibitors (0–330 nM or 0–1,000 nM) for 1 h. Following incubation, the substrate 3226-v was added to achieve a final concentration of 10 μM. The IC_50_ values are mean ± S.D. from three independent experiments.

For enzyme kinetic measurements of the inhibitors, 0.4 nM active MMP-9_Cat-H6 was incubated with various concentrations of inhibitors (0–25 nM) for 1 h. The reactions were initiated by the addition of substrate 3226-v at a final concentration of 10 μM. The inhibition constant (*K*_*i*_) values were determined by fitting the Morrison equation for tightly binding inhibitors [49]. Data were plotted as relative reaction velocity *versus* inhibitor concentration and fitted by nonlinear regression to Eq 1 using GraphPad Prism:

$$\frac{V}{V_{0}}=1-\frac{{\left[E\right]}_{t}+[I]+K_{i}^{\text{app}}-\sqrt{{\left({\left[E\right]}_{t}+[I]+K_{i}^{\text{app}}\right)}^{2}-4{\left[E\right]}_{t}[I]}}{2{\left[E\right]}_{t}}$$ (1)

$$K_{i}^{\text{app}}=K_{i}(1+\frac{\left[S\right]}{K_{m}})$$ (2)

where *V* is the initial reaction velocity; *V*_0_ is the initial reaction velocity in the absence of inhibitor; [*E*]_*t*_ is the total concentration of enzyme; [*I*] is the inhibitor concentration; [*S*] is the substrate concentration; *K*_*m*_ is the Michaelis-Menten constant; and *K*_*i*_^app^ is the apparent inhibition constant given by Eq 2. The *K*_*m*_ values were determined by measuring the initial reaction velocities of hydrolysis of 3226-v (2.5–10 μM) by active MMP-9_Cat-H6 [50]. Results are mean ± S.D. for three independent experiments.

For enzymatic assay with a macromolecular substrate, 0.6 nM active MMP-9_Cat-H6 was incubated with threefold serially diluted inhibitors (0–100 nM) for 1 h. Following incubation, DQ-gelatin was added to achieve a final concentration of 10 μg/ml. The IC_50_ values are mean ± S.D. for three independent experiments.

For specificity experiments, enzymatic assays using 12 human MMPs were carried out. Each active MMP was incubated with 1 μM inhibitors for 10 or 60 min, followed by the addition of each fluorescence-quenching peptide substrate. Data are expressed as the percentage of uninhibited activity. Experimental conditions of each MMP are described in S2 Table.

### Statistical analyses

All statistical analyses were performed using GraphPad Prism version 5.0 (GraphPad Software). Sample size for each experimental group was reported in the figure legends. The data are presented as mean ± S.D. One-way analysis of variance (ANOVA) followed by Dunnett’s test was used to determine the statistical significance in the comparison of IC50 value of the inhibitor against MMP-9 mutants *versus* wild-type MMP-9. *P* values less than 0.05 were considered statistical significant.

## Supporting information

S1 Fig SDS-PAGE analysis of purified MMP-9. (A) Pro-MMP-9_Cat-H6 was purified and activated by active MMP-3, as described in “Materials and methods.” (B) Pro-MMP-9_Cat_E402Q-H6 was purified and processed by active MMP-3, as described in “Materials and methods.” SDS-PAGE analysis of the purified pro-MMP-9 (1 μg per gel lane), active MMP-9_Cat-H6, and MMP-9_Cat_E402Q-H6 (0.5 μg per gel lane) was performed under reducing conditions followed by Coomassie Brilliant Blue G-250 staining.
(TIF)

S2 Fig Scheme of a randomized region of the engineered SPINK2. (A) Amino acid sequence of wild-type SPINK2. Lines indicate disulfide bonds (Cys-14–Cys-44, Cys-22–Cys-41, Cys-30–Cys-62). (B) Region randomized to create the engineered SPINK2 library. (C) Three-dimensional structure of wild-type SPINK2 (PDB code, 2JXD). SPINK2 is shown as a gray and red semi-transparent surface model; red indicates the randomized region. The right figure represents the left image turned 90° counterclockwise about the y-axis. (D) Aligned sequences of the engineered SPINK2-derived inhibitors against MMP-9.
(TIF)

S3 Fig The binding properties of inhibitors were evaluated by ELISA. Various concentrations of pro-MMP-9_Cat-Avi or active MMP-9_Cat-Avi (0.8–100 nM) were added to M91005 or GS-5745 (10 μg/ml each)-coated plates, and then C-terminal FLAG tag of captured MMP-9 was detected by HRP-conjugated anti-FLAG tag antibody. All curves were obtained by non-linear curve fitting.
(TIF)

S4 Fig Sequence alignment of pre-pro-form lacking the hemopexin domain of MMP-1, -2, -8, -9, and -13. The residues are numbered according to the generic MMP-9 nomenclature. Fn-like domain of MMP-2 and -9 is represented as XXX. Symbols denote catalytic glutamate residue (*asterisk*), and residues interacting with catalytic zinc ion (Z1), structural zinc ion (Z2), and calcium ions (Ca). Conserved residues among the five MMPs are shown with a *gray* background.
(TIF)

S5 Fig Purification and activation of MMP-9 mutants. (A) and (B) Pro-EK-MMP-9_Cat (WT), pro-forms of the cleft mutants, and exosite mutants were purified using HisTrap excel gel and gelatin-Sepharose resin. Each pro-MMP-9 (4 μM) was incubated with EKMax Enterokinase (32 U/ml) for 2 h at 4°C. After the activation of MMP-9, EK was removed by EKapture Agarose and buffer was exchanged for PBS at 4°C. SDS-PAGE analysis of the purified pro-MMP-9 (A, 1 μg per gel lane) and activated MMP-9 (B, 0.5 μg per gel lane, *asterisk* indicates 1 μg per gel lane) was performed under reducing conditions followed by Coomassie Brilliant Blue G-250 staining. All of the pro- and active MMP-9 mutants were highly purified. (C) MMP-9 activities of each mutant were determined by enzymatic assay using peptide substrate. Active MMP-9_Cat (WT) or activated mutants (1 nM each) were incubated with peptide substrate 3226-v (10 μM), and then MMP-9 activities were determined by monitoring the increase of fluorescence signal of the substrate. The activities of each mutant were normalized to that of WT. Most of the activated mutants, except for the L188A mutant, showed sufficient proteolytic activity to use for enzymatic assays. Each *bar* represents the mean ± S.D. (n = 3).
(TIF)

S6 Fig MMP-9 inhibitory activities of sc-311438 towards the cleft mutants (A) and the exosite mutants (B). All assay conditions and data presentation are the same as in Fig 5, and Tables 2 and 3. Statistical analysis of cleft mutants *versus* WT was performed by one-way ANOVA with Dunnett’s post tests for multiple comparisons.
(TIF)

S7 Fig MMP-9 inhibitory activities of M91005 towards three different MMP-9 constructs. For enzymatic assay with three different MMP-9 constructs, namely, full-length MMP-9, the form with HPX domain deleted (MMP-9_Cat), and the form with both Fn-like domain and HPX domain deleted (the catalytic domain), 0.4 nM active MMP-9 was incubated with threefold serially diluted inhibitors (0–100 nM) for 1 h. Following incubation, substrate 3226-v was added to achieve a final concentration of 10 μM. The other assay conditions are the same as in Fig 1C.
(TIF)

S8 Fig Expression of active-site mutants of MMP-9 (H401A, H405A, and H411A). Western blot analysis of the culture supernatants (6.5 μl per gel lane) of HEK293F cells transfected with each MMP-9 expression vector was performed under reducing conditions. After electrophoresis, the proteins were transferred to a PVDF membrane followed by blocking with 5% skim milk in PBS-T. After washing with PBS-T, Penta His HRP Conjugate (QIAGEN, 34460) was added (1:10,000 dilution in PBS-T with 0.5% skim milk) and incubated for 1 h at room temperature. After washing with PBS-T, the reaction was developed with ECL Prime Western Blotting Detection Reagent (GE Healthcare) at room temperature. The pre-stained visible protein markers and the chemiluminescent signals were captured using a ChemiDoc XRS+ CCD camera-based imager system (Bio-Rad). *Black arrowhead* indicates the band of full-length MMP-9 fused to a C-terminal His_6_ tag.
(TIF)

S9 Fig C-terminal degradation of MMP-9 during activation reaction using trypsin. For MMP-9 activation using trypsin, the final 4 μM pro-MMP-9_Cat-H6 was incubated with 2.7 μM TPCK trypsin (Thermo Fisher Scientific, 20233) in TNC buffer at 37°C. Western blot analysis of the activation reaction solution (0.6 μg of MMP-9 per gel lane) was performed under reducing conditions. The experimental conditions after electrophoresis were carried out according to S8 Fig. *Black arrowhead* and *red arrowhead* indicate the band of the product obtained by the activation reaction using trypsin and the band of C-terminal fragment of pro-MMP-9_Cat-H6, respectively.
(TIF)

S10 Fig Autolysis of active MMP-9 upon long-term incubation at 25°C. To evaluate the stability of the bait protein, 2.4 μM of biotinylated active MMP-9_Cat-BAP was incubated in PBS at 25°C for 16 h. SDS-PAGE analysis of the biotinylated active MMP-9_Cat-BAP after incubation (0.5 μg per gel lane) was performed under reducing conditions followed by Coomassie Brilliant Blue G-250 staining. *Black arrowhead* and *red arrowhead* indicate the band of the biotinylated active MMP-9_Cat-BAP and the band of degradation product, respectively.
(TIF)

S1 Table MMP inhibitory activities of inhibitors against eight MMPs. The cross-reactivities against MMP-3, -7, -10, -12, -14, -15, -16, and -17 were measured by enzymatic assay using peptide substrate. Each active MMP was incubated with inhibitors (1 μM), and then the peptide substrate (10 μM 3168-v for MMP-3, 10 μM 3226-v for the other MMPs) was added as described under “Materials and methods.” Enzymatic activity was determined by monitoring the hydrolysis of the peptide substrate and each remaining enzymatic activity was normalized to the activity in the absence of inhibitors. Data are shown as the mean ± S.D. (n = 3).
(DOCX)

S2 Table MMP enzymatic assay conditions. (DOCX)

S3 Table PCR primers used for site-directed mutagenesis. (DOCX)

S1 Protocol Protocol for ELISA assay using anti-MMP-9 antibody GS-5745. (DOCX)

S1 Raw images The original uncropped and unadjusted image to prepare the final figures. Images were captured using a ChemiDocXRS+ CCD camera-based imager system (Bio-Rad). The part of the blot or gel shown in the final figure is within the red box. Lanes marked with an "X" were not included in the final figure.
(PDF)
