# Supplementary figures and images for: Discovery of potent and specific inhibitors targeting the active site of MMP-9 from the engineered SPINK2 library
Source: PLoS One. 2020 Dec 29;15(12):e0244656. doi: 10.1371/journal.pone.0244656 (PMC7771667; doi:10.1371/journal.pone.0244656)

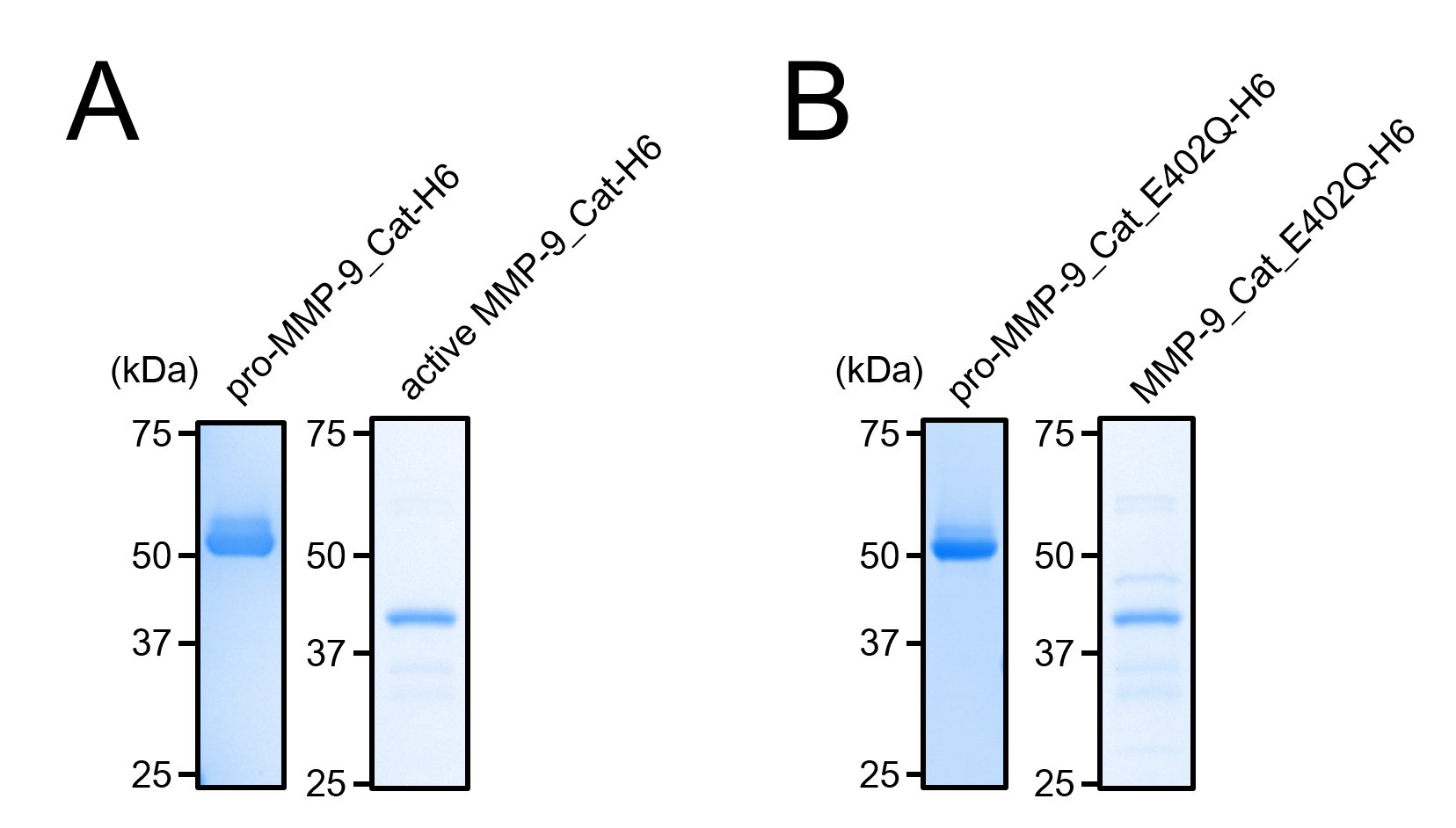

Supplement: S1 Fig — (A) Pro-MMP-9_Cat-H6 was purified and activated by active MMP-3, as described in “Materials and methods.” (B) Pro-MMP-9_Cat_E402Q-H6 was purified and processed by active MMP-3, as described in “Materials and methods.” SDS-PAGE analysis of the purified pro-MMP-9 (1 μg per gel lane), active MMP-9_Cat-H6, and MMP-9_Cat_E402Q-H6 (0.5 μg per gel lane) was performed under reducing conditions followed by Coomassie Brilliant Blue G-250 staining. (TIF) [file pone.0244656.s001.tif]

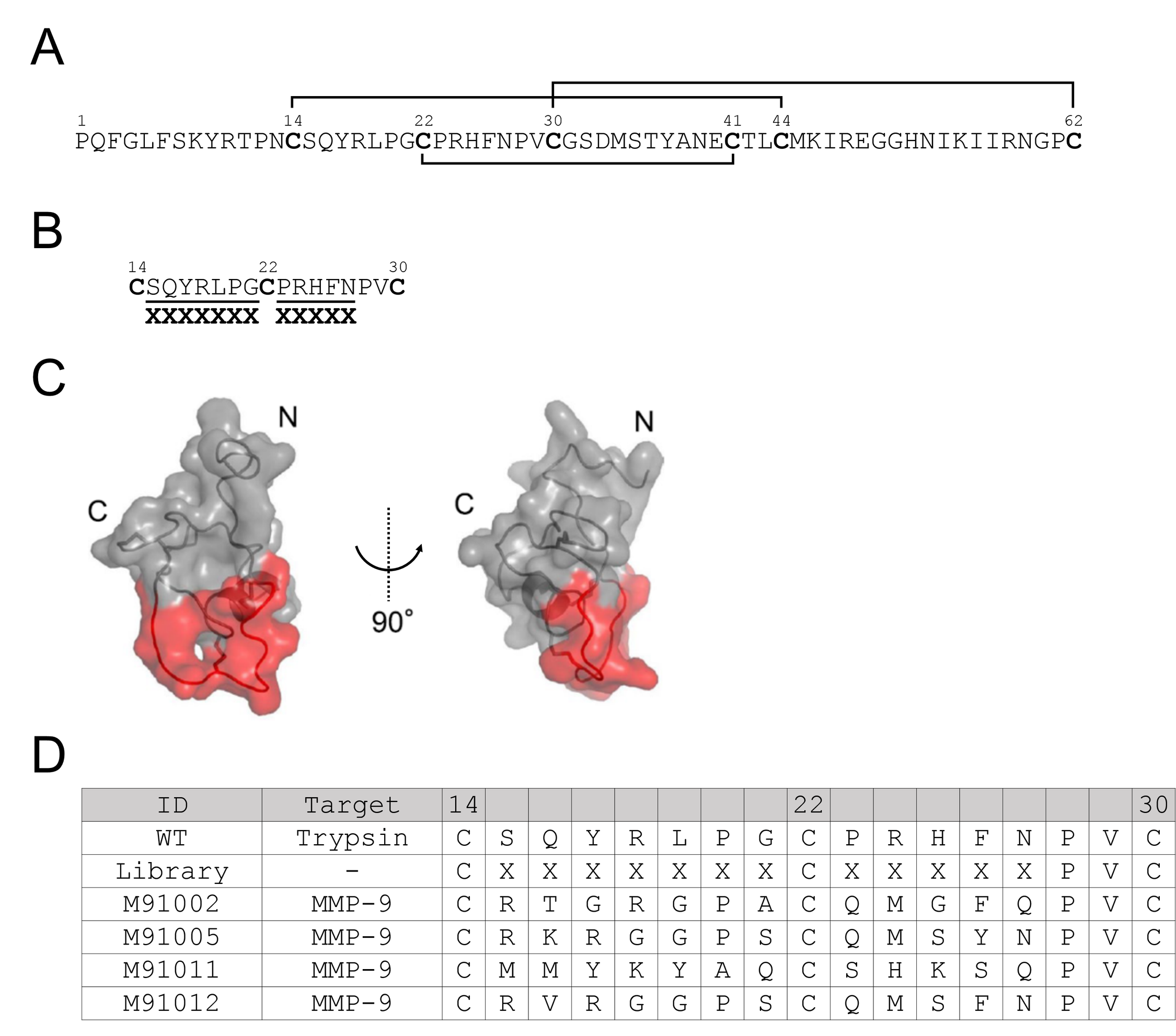

Supplement: S2 Fig — (A) Amino acid sequence of wild-type SPINK2. Lines indicate disulfide bonds (Cys-14–Cys-44, Cys-22–Cys-41, Cys-30–Cys-62). (B) Region randomized to create the engineered SPINK2 library. (C) Three-dimensional structure of wild-type SPINK2 (PDB code, 2JXD). SPINK2 is shown as a gray and red semi-transparent surface model; red indicates the randomized region. The right figure represents the left image turned 90° counterclockwise about the y-axis. (D) Aligned sequences of the engineered SPINK2-derived inhibitors against MMP-9. (TIF) [file pone.0244656.s002.tif]

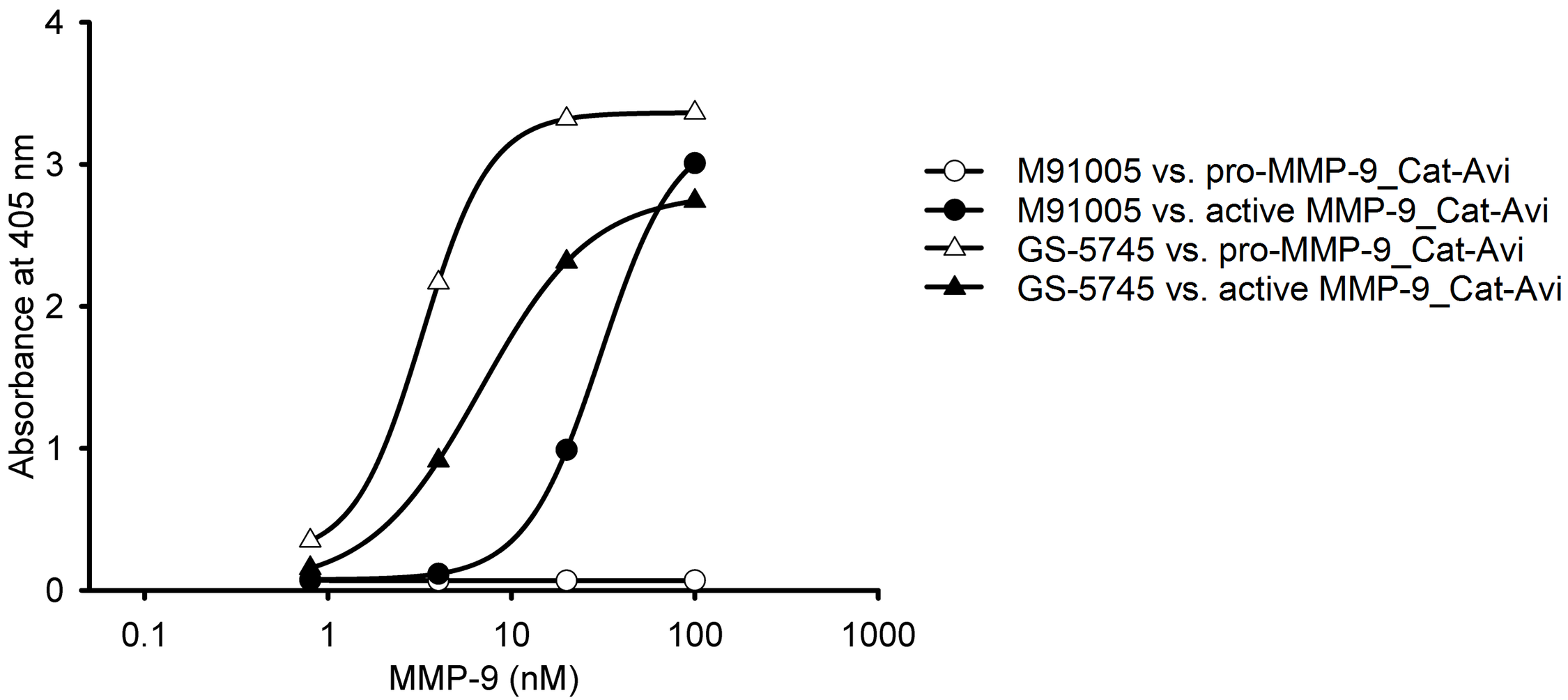

Supplement: S3 Fig — Various concentrations of pro-MMP-9_Cat-Avi or active MMP-9_Cat-Avi (0.8–100 nM) were added to M91005 or GS-5745 (10 μg/ml each)-coated plates, and then C-terminal FLAG tag of captured MMP-9 was detected by HRP-conjugated anti-FLAG tag antibody. All curves were obtained by non-linear curve fitting. (TIF) [file pone.0244656.s003.tif]

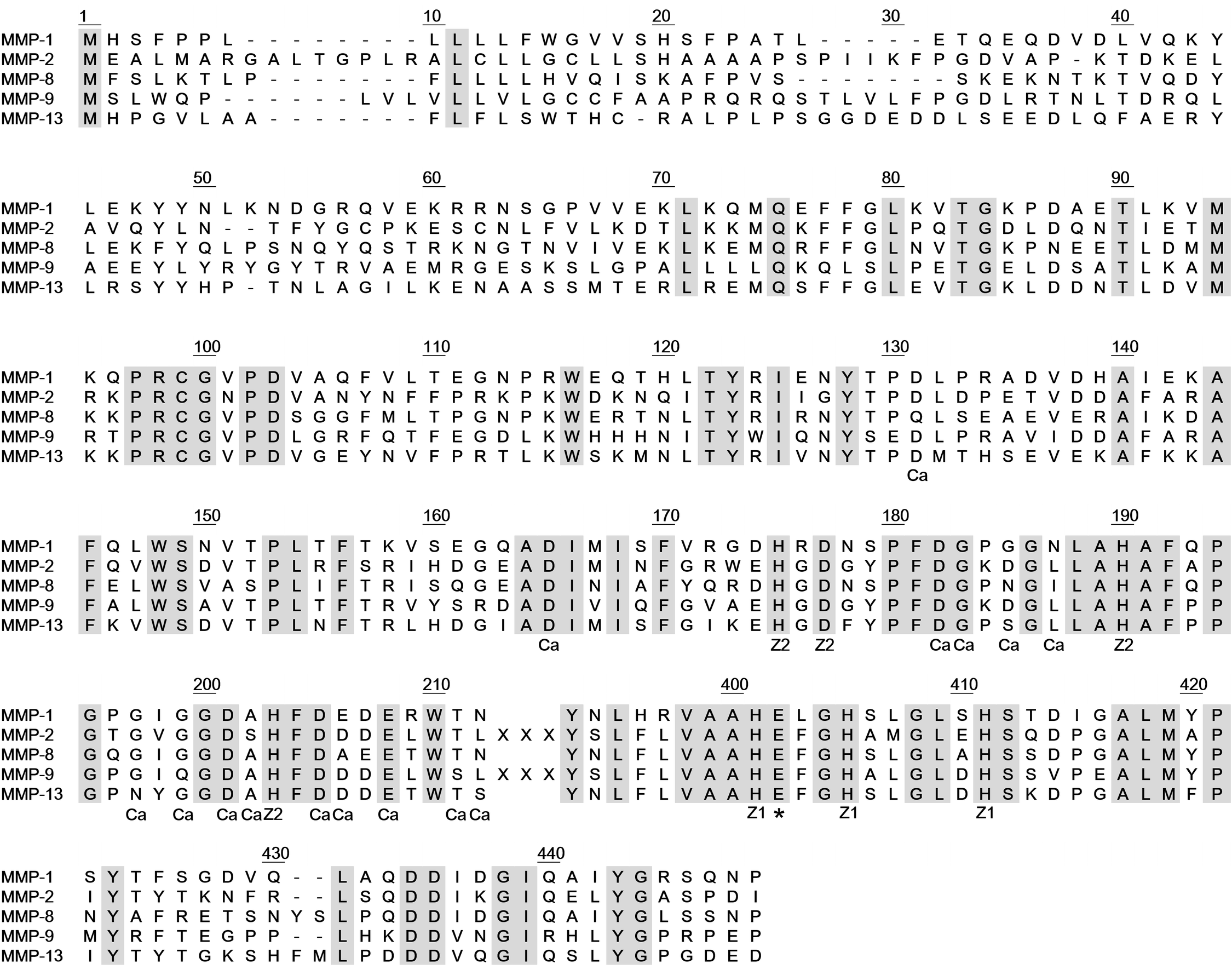

Supplement: S4 Fig — The residues are numbered according to the generic MMP-9 nomenclature. Fn-like domain of MMP-2 and -9 is represented as XXX. Symbols denote catalytic glutamate residue (asterisk), and residues interacting with catalytic zinc ion (Z1), structural zinc ion (Z2), and calcium ions (Ca). Conserved residues among the five MMPs are shown with a gray background. (TIF) [file pone.0244656.s004.tif]

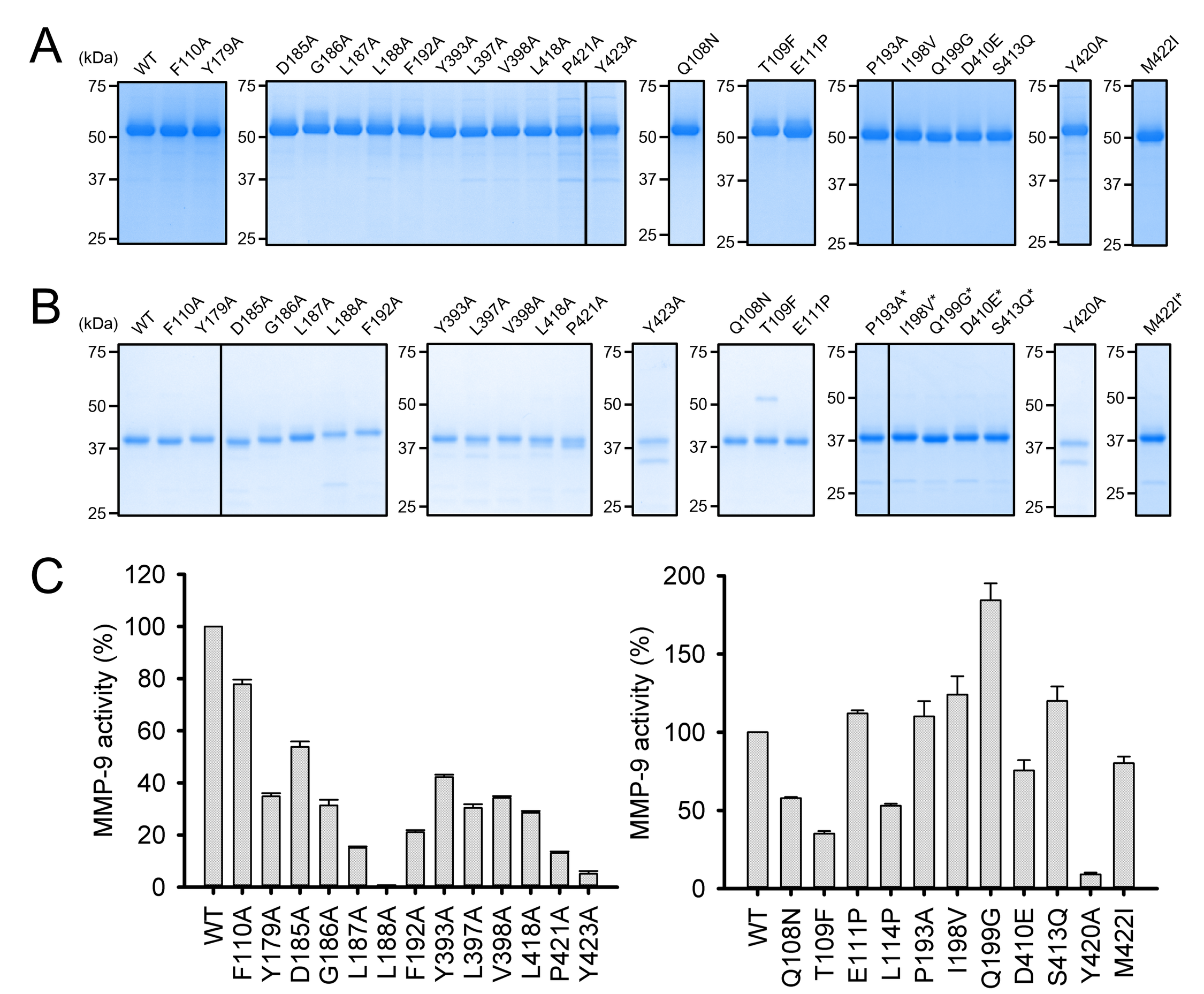

Supplement: S5 Fig — (A) and (B) Pro-EK-MMP-9_Cat (WT), pro-forms of the cleft mutants, and exosite mutants were purified using HisTrap excel gel and gelatin-Sepharose resin. Each pro-MMP-9 (4 μM) was incubated with EKMax Enterokinase (32 U/ml) for 2 h at 4°C. After the activation of MMP-9, EK was removed by EKapture Agarose and buffer was exchanged for PBS at 4°C. SDS-PAGE analysis of the purified pro-MMP-9 (A, 1 μg per gel lane) and activated MMP-9 (B, 0.5 μg per gel lane, asterisk indicates 1 μg per gel lane) was performed under reducing conditions followed by Coomassie Brilliant Blue G-250 staining. All of the pro- and active MMP-9 mutants were highly purified. (C) MMP-9 activities of each mutant were determined by enzymatic assay using peptide substrate. Active MMP-9_Cat (WT) or activated mutants (1 nM each) were incubated with peptide substrate 3226-v (10 μM), and then MMP-9 activities were determined by monitoring the increase of fluorescence signal of the substrate. The activities of each mutant were normalized to that of WT. Most of the activated mutants, except for the L188A mutant, showed sufficient proteolytic activity to use for enzymatic assays. Each bar represents the mean ± S.D. (n = 3). (TIF) [file pone.0244656.s005.tif]

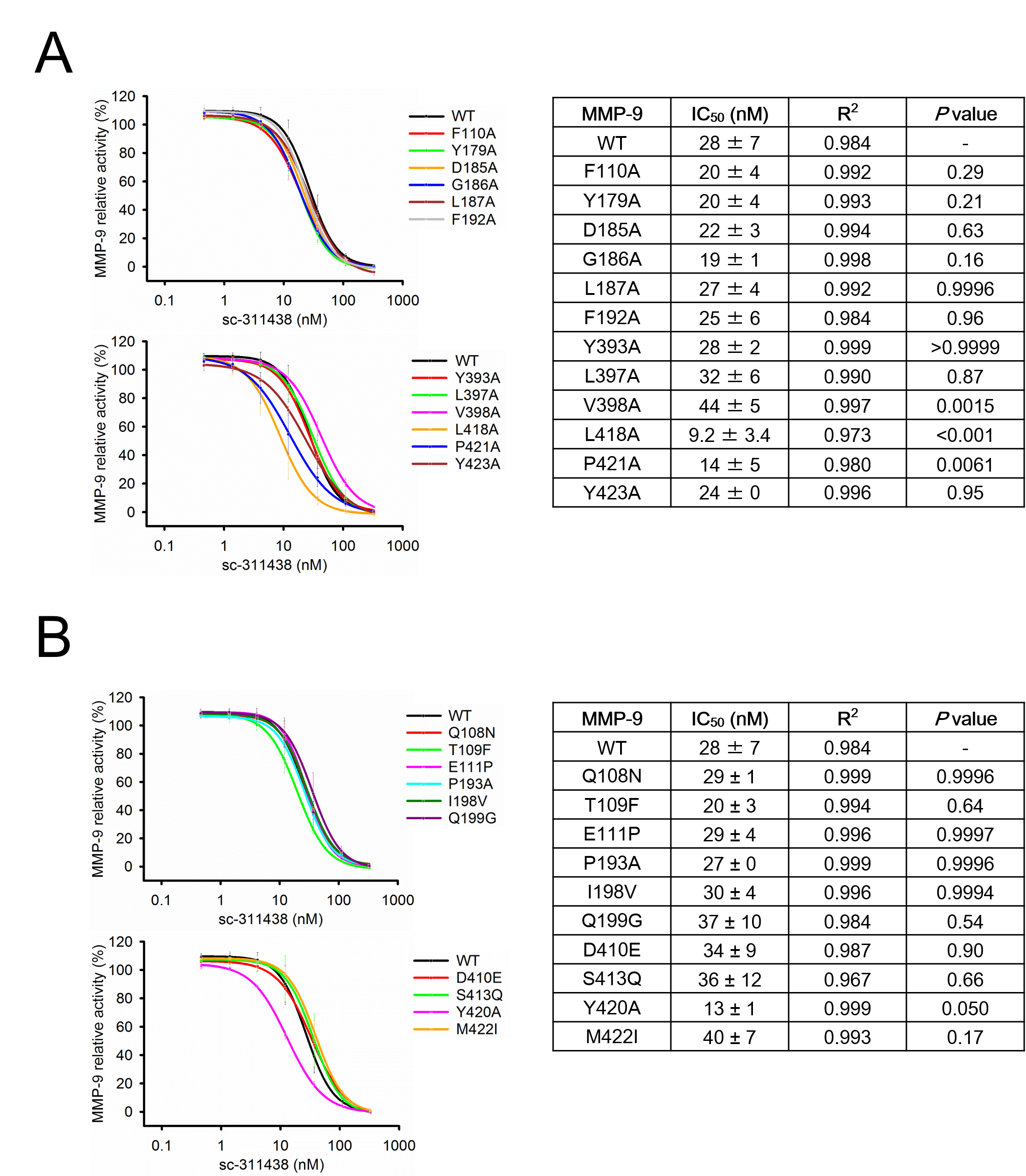

Supplement: S6 Fig — All assay conditions and data presentation are the same as in Fig 5, and Tables 2 and 3. Statistical analysis of cleft mutants versus WT was performed by one-way ANOVA with Dunnett’s post tests for multiple comparisons. (TIF) [file pone.0244656.s006.tif]

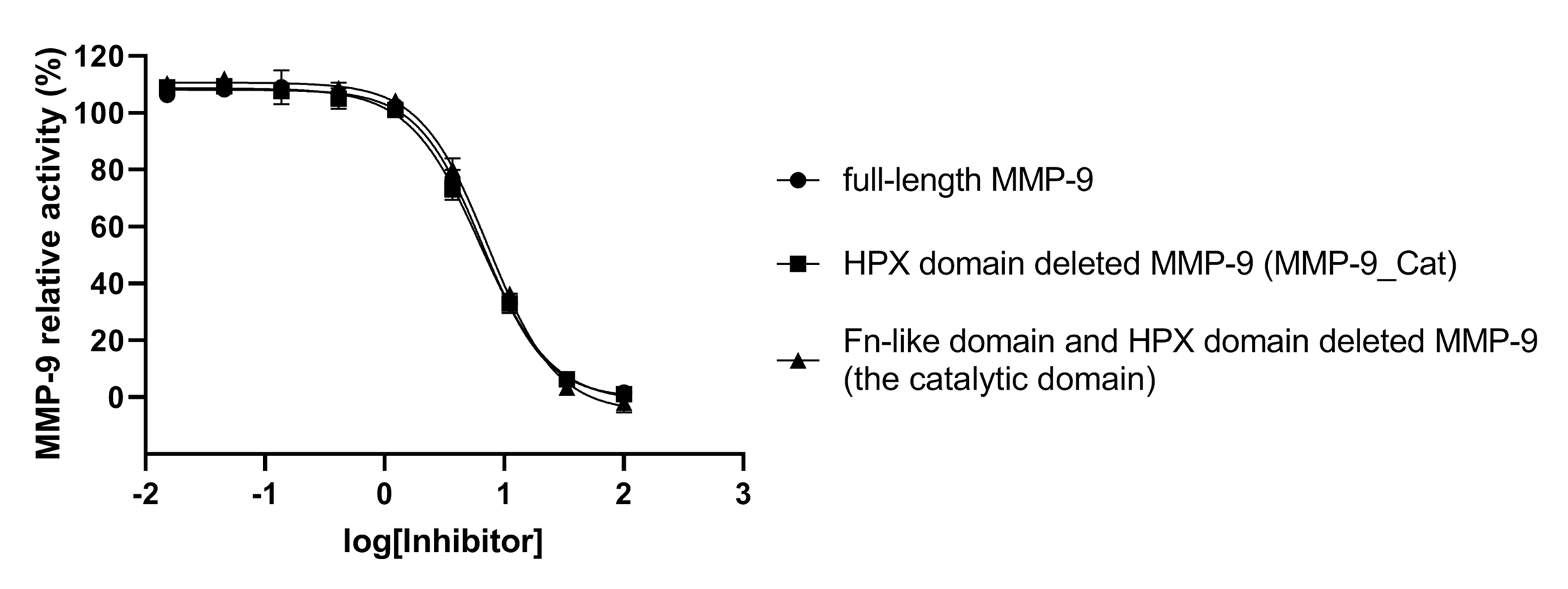

Supplement: S7 Fig — For enzymatic assay with three different MMP-9 constructs, namely, full-length MMP-9, the form with HPX domain deleted (MMP-9_Cat), and the form with both Fn-like domain and HPX domain deleted (the catalytic domain), 0.4 nM active MMP-9 was incubated with threefold serially diluted inhibitors (0–100 nM) for 1 h. Following incubation, substrate 3226-v was added to achieve a final concentration of 10 μM. The other assay conditions are the same as in Fig 1C. (TIF) [file pone.0244656.s007.tif]

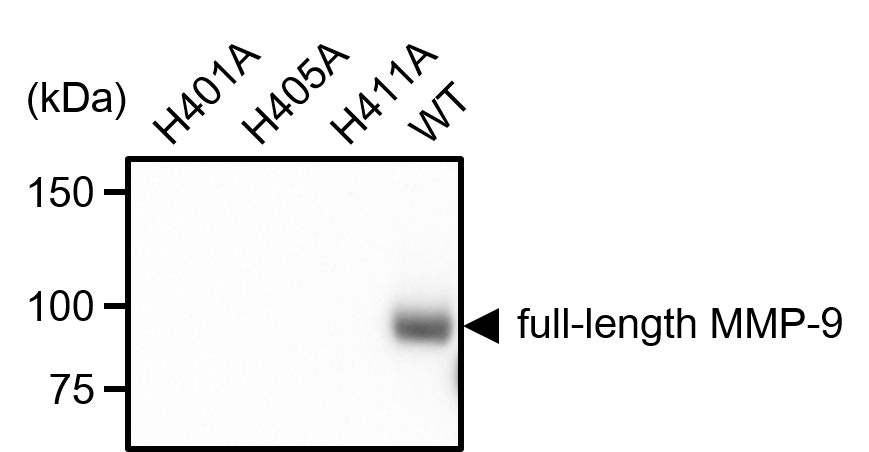

Supplement: S8 Fig — Western blot analysis of the culture supernatants (6.5 μl per gel lane) of HEK293F cells transfected with each MMP-9 expression vector was performed under reducing conditions. After electrophoresis, the proteins were transferred to a PVDF membrane followed by blocking with 5% skim milk in PBS-T. After washing with PBS-T, Penta His HRP Conjugate (QIAGEN, 34460) was added (1:10,000 dilution in PBS-T with 0.5% skim milk) and incubated for 1 h at room temperature. After washing with PBS-T, the reaction was developed with ECL Prime Western Blotting Detection Reagent (GE Healthcare) at room temperature. The pre-stained visible protein markers and the chemiluminescent signals were captured using a ChemiDoc XRS+ CCD camera-based imager system (Bio-Rad). Black arrowhead indicates the band of full-length MMP-9 fused to a C-terminal His6 tag. (TIF) [file pone.0244656.s008.tif]

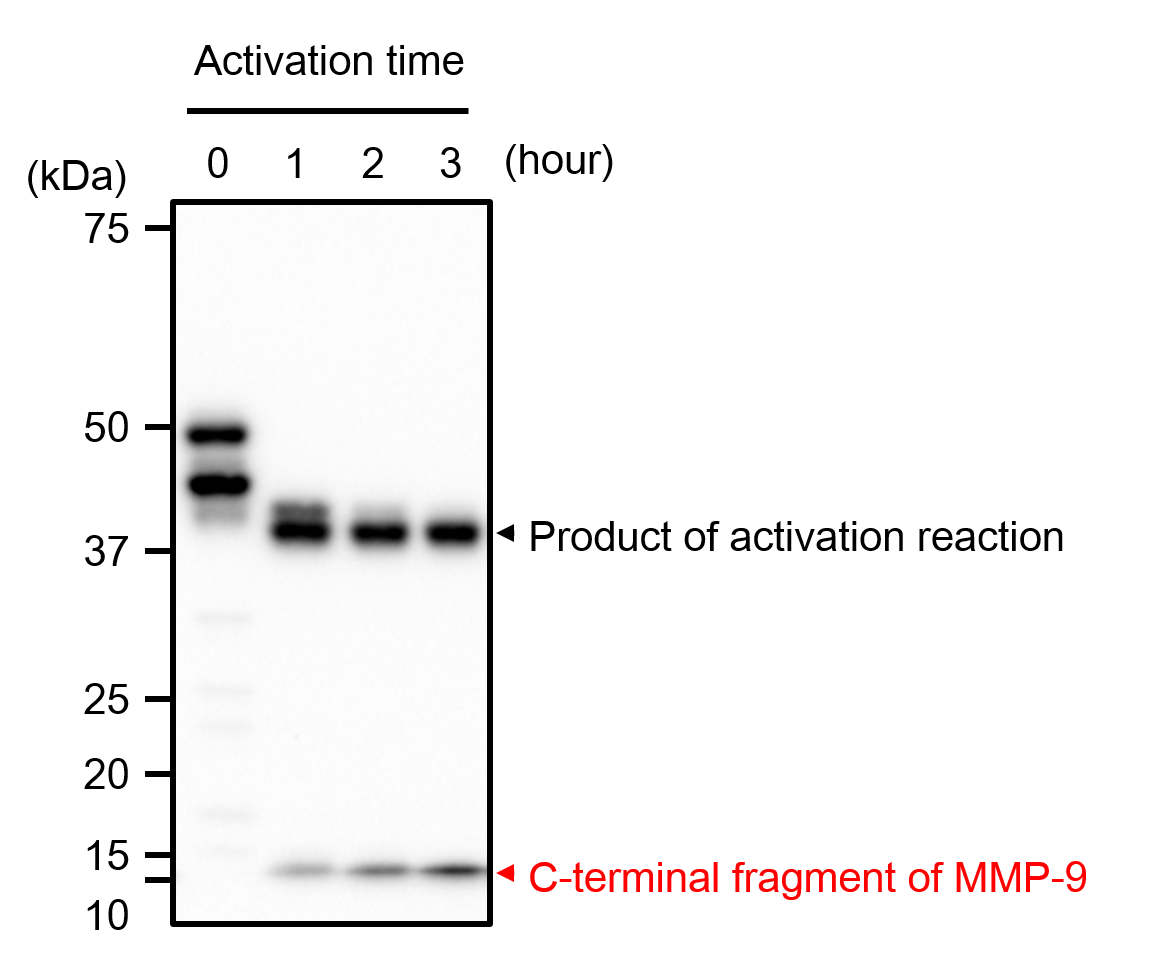

Supplement: S9 Fig — For MMP-9 activation using trypsin, the final 4 μM pro-MMP-9_Cat-H6 was incubated with 2.7 μM TPCK trypsin (Thermo Fisher Scientific, 20233) in TNC buffer at 37°C. Western blot analysis of the activation reaction solution (0.6 μg of MMP-9 per gel lane) was performed under reducing conditions. The experimental conditions after electrophoresis were carried out according to S8 Fig. Black arrowhead and red arrowhead indicate the band of the product obtained by the activation reaction using trypsin and the band of C-terminal fragment of pro-MMP-9_Cat-H6, respectively. (TIF) [file pone.0244656.s009.tif]

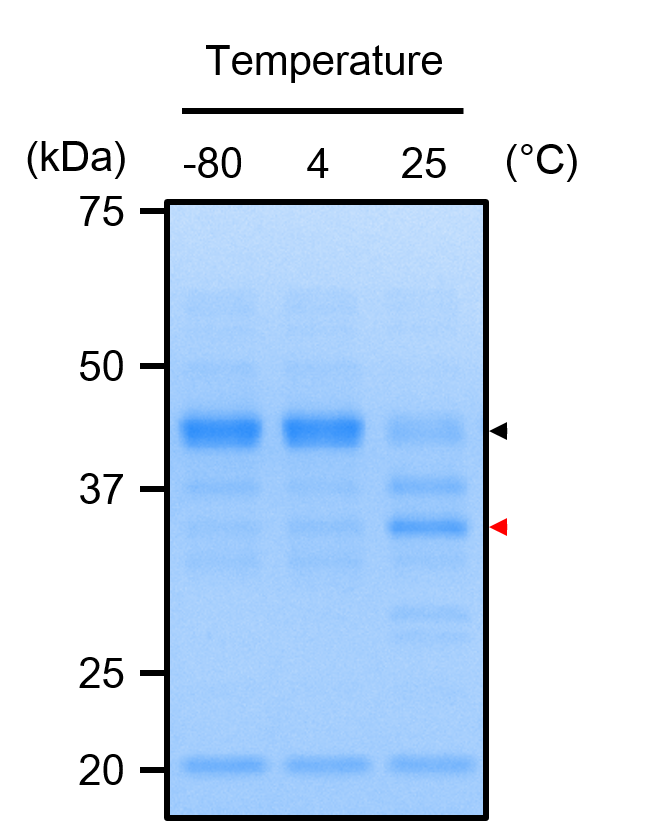

Supplement: S10 Fig — To evaluate the stability of the bait protein, 2.4 μM of biotinylated active MMP-9_Cat-BAP was incubated in PBS at 25°C for 16 h. SDS-PAGE analysis of the biotinylated active MMP-9_Cat-BAP after incubation (0.5 μg per gel lane) was performed under reducing conditions followed by Coomassie Brilliant Blue G-250 staining. Black arrowhead and red arrowhead indicate the band of the biotinylated active MMP-9_Cat-BAP and the band of degradation product, respectively. (TIF) [file pone.0244656.s010.tif]
