# Supplementary material for: Discovery of potent and specific inhibitors targeting the active site of MMP-9 from the engineered SPINK2 library
Source: PLoS One. 2020 Dec 29;15(12):e0244656. doi: 10.1371/journal.pone.0244656 (PMC7771667; doi:10.1371/journal.pone.0244656)
Supplement: S1 Table — The cross-reactivities against MMP-3, -7, -10, -12, -14, -15, -16, and -17 were measured by enzymatic assay using peptide substrate. Each active MMP was incubated with inhibitors (1 μM), and then the peptide substrate (10 μM 3168-v for MMP-3, 10 μM 3226-v for the other MMPs) was added as described under “Materials and methods.” Enzymatic activity was determined by monitoring the hydrolysis of the peptide substrate and each remaining enzymatic activity was normalized to the activity in the absence of inhibitors. Data are shown as the mean ± S.D. (n = 3). (DOCX) [file pone.0244656.s011.docx]

| Enzyme | Remaining enzymatic activity with inhibitor | | | | |
| --- | --- | --- | --- | --- | --- |
|  | M91002 | M91005 | M91011 | M91012 | sc-311438 |
| MMP-3 | 116 ± 6% | 114 ± 4% | 115 ± 3% | 118 ± 2% | 1.6 ± 1.5% |
| MMP-7 | 121 ± 1% | 123 ± 2% | 124 ± 1% | 120 ± 4% | 45 ± 2% |
| MMP-10 | 144 ± 13% | 141 ± 9% | 159 ± 26% | 144 ± 11% | 0% |
| MMP-12 | 125 ± 18% | 122 ± 18% | 124 ± 21% | 123 ± 20% | 0% |
| MMP-14 | 115 ± 4% | 112 ± 6% | 114 ± 8% | 112 ± 7% | 0% |
| MMP-15 | 121 ± 11% | 117 ± 9% | 115 ± 13% | 115 ± 11% | 0% |
| MMP-16 | 110 ± 7% | 110 ± 8% | 117 ± 7% | 107 ± 6% | 3.0 ± 0.1% |
| MMP-17 | 124 ± 7% | 127 ± 2% | 132 ± 16% | 132 ± 18% | 0% |
