# Supplementary material for: Discovery of potent and specific inhibitors targeting the active site of MMP-9 from the engineered SPINK2 library
Source: PLoS One. 2020 Dec 29;15(12):e0244656. doi: 10.1371/journal.pone.0244656 (PMC7771667; doi:10.1371/journal.pone.0244656)
Supplement: S2 Table — (DOCX) [file pone.0244656.s012.docx]

| Enzyme | Final concentration of enzyme (nM) | Pre-incubation time (min) | Substrate | Final concentration of substrate (µM) |
| --- | --- | --- | --- | --- |
| MMP-1 | 5 | 60 | 3226-v | 10 |
| MMP-2 | 1.4 | 60 | 3226-v | 50 |
| MMP-3 | 25 | 60 | 3168-v | 10 |
| MMP-7 | 1 | 60 | 3226-v | 10 |
| MMP-8 | 0.6 | 60 | 3226-v | 10 |
| MMP-10 | 4 | 60 | 3226-v | 10 |
| MMP-12 | 4 | 60 | 3226-v | 10 |
| MMP-13 | 2 | 10 | 3226-v | 10 |
| MMP-14 | 1 | 60 | 3226-v | 10 |
| MMP-15 | 1 | 60 | 3226-v | 10 |
| MMP-16 | 3 | 60 | 3226-v | 10 |
| MMP-17 | 3 | 10 | 3226-v | 10 |
