# Supplementary material for: Discovery of potent and specific inhibitors targeting the active site of MMP-9 from the engineered SPINK2 library
Source: PLoS One. 2020 Dec 29;15(12):e0244656. doi: 10.1371/journal.pone.0244656 (PMC7771667; doi:10.1371/journal.pone.0244656)
Supplement: S3 Table — (DOCX) [file pone.0244656.s013.docx]

| Mutant | Primers (5’ - 3’) | |
| --- | --- | --- |
| pro-MMP-9_Cat_E402Q-H6 | Forward: | TTCCTGGTGGCCGCCCACCAGTTTGGACACGCCCTGGGC |
|  | Reverse: | GCCCAGGGCGTGTCCAAACTGGTGGGCGGCCACCAGGAA |
| pro-EK-MMP-9_Cat_F110A | Forward: | GCCGAGGGCGATCTGAAGTGGCAC |
|  | Reverse: | GGTCTGGAATTTATCATCGTCATC |
| pro-EK-MMP-9_Cat_Y179A | Forward: | GCTCCTTTCGATGGCAAGGATGGACTG |
|  | Reverse: | GCCGTCGCCGTGTTCGGCCACGCC |
| pro-EK-MMP-9_Cat_D185A | Forward: | GCCGGACTGCTGGCCCACGCCTTTCC |
|  | Reverse: | CTTGCCATCGAAAGGGTAGCCGTC |
| pro-EK-MMP-9_Cat_G186A | Forward: | GCTCTGCTGGCCCACGCCTTTCCAC |
|  | Reverse: | ATCCTTGCCATCGAAAGGGTAGCC |
| pro-EK-MMP-9_Cat_L187A | Forward: | GCTCTGGCCCACGCCTTTCCACCTGG |
|  | Reverse: | TCCATCCTTGCCATCGAAAGGGTAGC |
| pro-EK-MMP-9_Cat_L188A | Forward: | GCTGCCCACGCCTTTCCACCTGGC |
|  | Reverse: | CAGTCCATCCTTGCCATCGAAAGG |
| pro-EK-MMP-9_Cat_F192A | Forward: | GCTCCACCTGGCCCTGGCATTCAG |
|  | Reverse: | GGCGTGGGCCAGCAGTCCATCCTTGC |
| pro-EK-MMP-9_Cat_Y393A | Forward: | GCCAGCCTGTTCCTGGTGGCC |
|  | Reverse: | GCCCTGGTCGGGACAGAAGCCCCAC |
| pro-EK-MMP-9_Cat_L397A | Forward: | GCCGTGGCCGCCCACGAATTTGGAC |
|  | Reverse: | GAACAGGCTGTAGCCCTGGTCG |
| pro-EK-MMP-9_Cat_V398A | Forward: | GCTGCCGCCCACGAATTTGGACAC |
|  | Reverse: | CAGGAACAGGCTGTAGCCCTGGTC |
| pro-EK-MMP-9_Cat_L418A | Forward: | GCTATGTACCCCATGTACCGGTTC |
|  | Reverse: | GGCCTCAGGCACAGAGCTGTGATC |
| pro-EK-MMP-9_Cat_P421A | Forward: | GCCATGTACCGGTTCACCGAGG |
|  | Reverse: | GTACATCAGGGCCTCAGGCACAGAG |
| pro-EK-MMP-9_Cat_Y423A | Forward: | GCCCGGTTCACCGAGGGCCCACC |
|  | Reverse: | CATGGGGTACATCAGGGCCTCAGG |
| pro-EK-MMP-9_Cat_Q108N | Forward: | AACACCTTCGAGGGCGATCTGAAG |
|  | Reverse: | GAATTTATCATCGTCATCTCTGCC |
| pro-EK-MMP-9_Cat_T109F | Forward: | TTCGAGGGCGATCTGAAGTGGCAC |
|  | Reverse: | GAACTGGAATTTATCATCGTCATC |
| pro-EK-MMP-9_Cat_E111P | Forward: | CCTGGCGATCTGAAGTGGCACCAC |
|  | Reverse: | GAAGGTCTGGAATTTATCATCGTC |
| pro-EK-MMP-9_Cat_P193A | Forward: | GCCCCTGGCCCTGGCATTCAGGGC |
|  | Reverse: | AAAGGCGTGGGCCAGCAGTCCATC |
| pro-EK-MMP-9_Cat_I198V | Forward: | GTGCAGGGCGACGCCCACTTCGAC |
|  | Reverse: | GCCAGGGCCAGGTGGAAAGGCGTG |
| pro-EK-MMP-9_Cat_Q199G | Forward: | GGCGGCGACGCCCACTTCGACGAC |
|  | Reverse: | AATGCCAGGGCCAGGTGGAAAGGC |
| pro-EK-MMP-9_Cat_D410E | Forward: | GAACACAGCTCTGTGCCTGAGGCC |
|  | Reverse: | CAGGCCCAGGGCGTGTCCAAATTC |
| pro-EK-MMP-9_Cat_S413Q | Forward: | CAGGTGCCTGAGGCCCTGATGTAC |
|  | Reverse: | GCTGTGATCCAGGCCCAGGGCGTG |
| pro-EK-MMP-9_Cat_Y420A | Forward: | GCTCCCATGTACCGGTTCACCGAG |
|  | Reverse: | CATCAGGGCCTCAGGCACAGAGCT |
| pro-EK-MMP-9_Cat_M422I | Forward: | ATTTACCGGTTCACCGAGGGCCCA |
|  | Reverse: | GGGGTACATCAGGGCCTCAGGCAC |
