# Supplementary material for: Discovery of potent and specific inhibitors targeting the active site of MMP-9 from the engineered SPINK2 library
Source: PLoS One. 2020 Dec 29;15(12):e0244656. doi: 10.1371/journal.pone.0244656 (PMC7771667; doi:10.1371/journal.pone.0244656)
Supplement: S1 Protocol — (DOCX) [file pone.0244656.s014.docx]

**Antibody expression and purification**

The DNA sequence encoding anti-MMP-9 antibody GS-5745, which was described in the patent WO2013/130078A1, was synthesized (Thermo Fisher Scientific). The heavy-chain and light-chain sequences of GS-5745 were cloned into pcDNA3.3 vector. They were co-expressed in HEK293F cells and then GS-5745 was purified using MabSelect SuRe resin (GE Healthcare), followed by buffer exchange into PBS.

**ELISA**

The inhibitor (10 µg/ml) in PBS was coated on a 96-well Nunc MaxiSorp plate (Thermo Fisher Scientific) overnight at 4°C. The plate was washed with PBS-T and blocked with 5% skim milk in PBS-T for 2 h at room temperature. Fivefold serially diluted pro-MMP-9_Cat-BAP or active MMP-9_Cat-BAP (0.8–100 nM) in PBS-T was added and incubated for 2 h at room temperature. After washing with PBS-T, HRP-conjugated anti-FLAG tag antibody (Sigma-Aldrich) was added (1:2,500 dilution in PBS-T) and incubated for 1 h at room temperature. After washing with PBS-T, the reaction was developed with 2,2′-azino-di-(3-ethylbenzthiazoline sulfonic acid) substrate at room temperature. The absorbance at 405 nm was measured using an EnSpire fluorescence plate reader.
