# Supplementary material for: Discovery of potent and specific inhibitors targeting the active site of MMP-9 from the engineered SPINK2 library
Source: PLoS One. 2020 Dec 29;15(12):e0244656. doi: 10.1371/journal.pone.0244656 (PMC7771667; doi:10.1371/journal.pone.0244656)
Supplement: S1 Raw images — Images were captured using a ChemiDocXRS+ CCD camera-based imager system (Bio-Rad). The part of the blot or gel shown in the final figure is within the red box. Lanes marked with an "X" were not included in the final figure. (PDF) [file pone.0244656.s015.pdf]

S1_raw_images: The original uncropped and unadjusted image to prepare the final figures. Images were captured using a ChemiDoc XRS+ CCD camera-based imager system (Bio-Rad). The part of the blot or gel shown in the final figure is within the red box. Lanes marked with an "X" were not included in the final figure.

Raw image for S1A Fig: SDS-PAGE analysis of purified MMP-9.

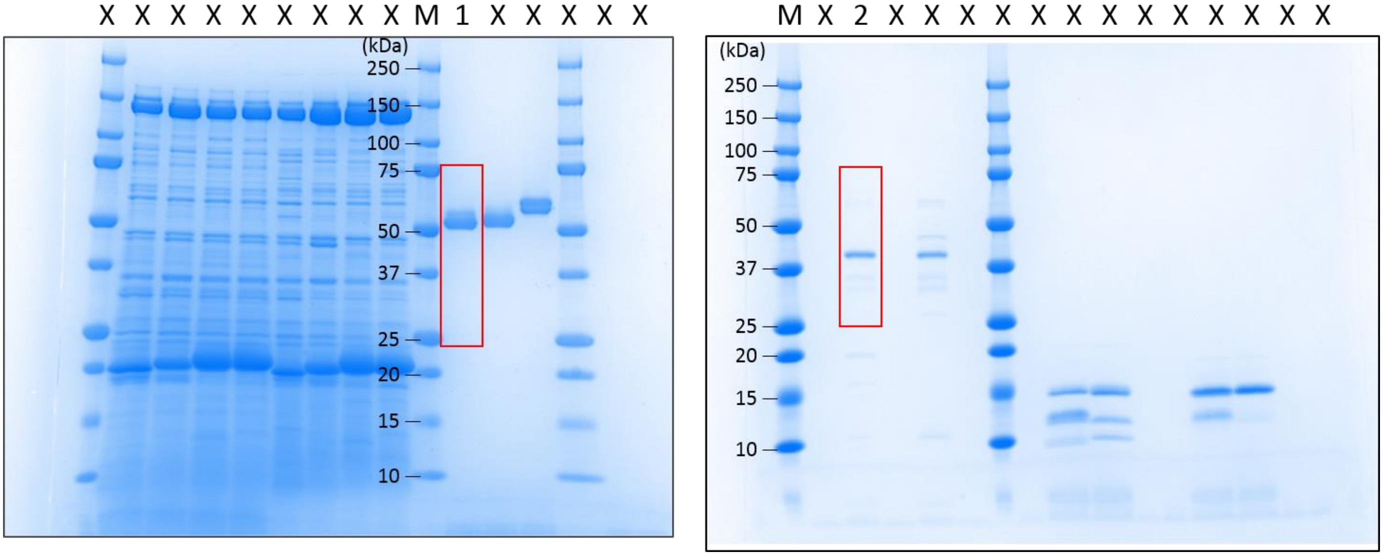

M. Precision Plus Protein Dual Color Standards (Bio-Rad, 161-0374)
1. pro-MMP-9_Cat-H6
2. active MMP-9_Cat-H6

Raw image for S1B Fig: SDS-PAGE analysis of purified MMP-9.

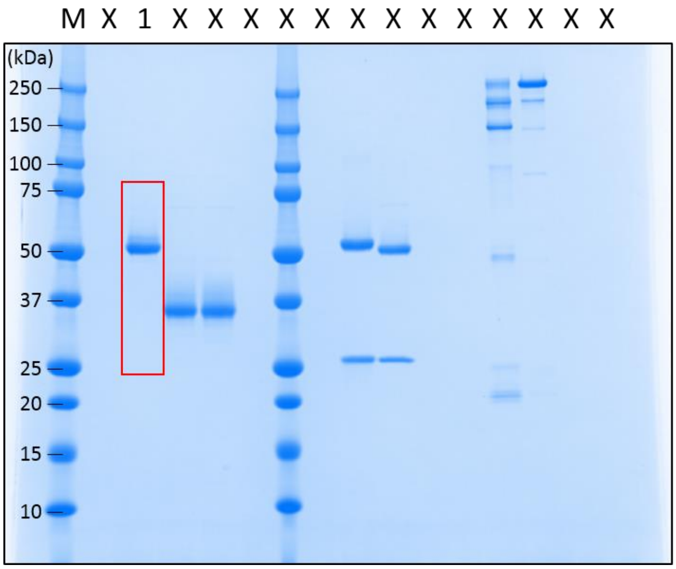

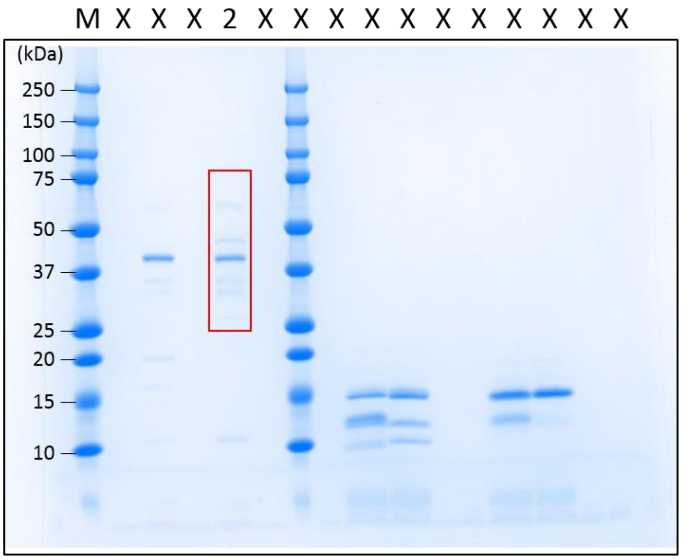

M. Precision Plus Protein Dual Color Standards (Bio-Rad, 161-0374)
1. pro-MMP-9_Cat_E402Q-H6
2. MMP-9_Cat_E402Q-H6

Raw image for S5A Fig: Purification and activation of MMP-9 mutants.

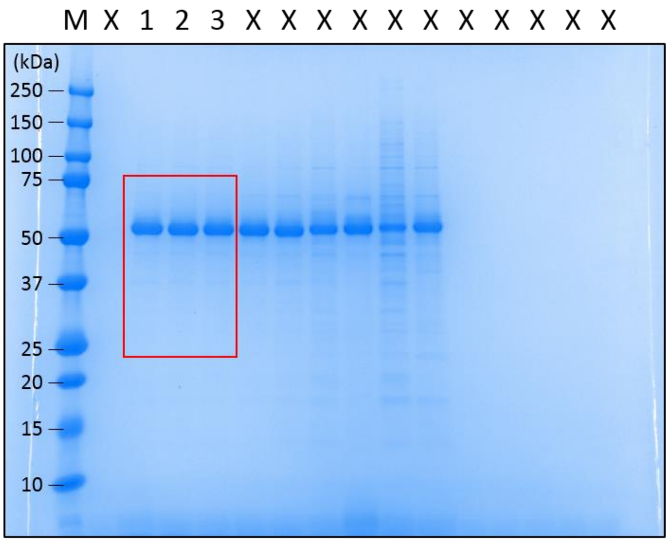

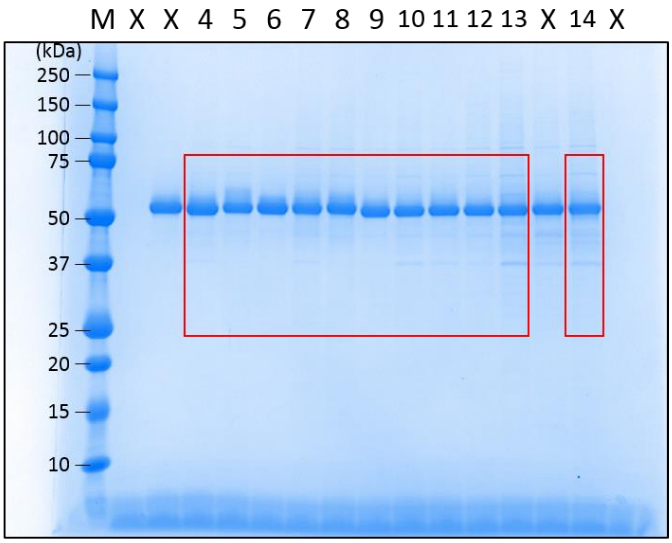

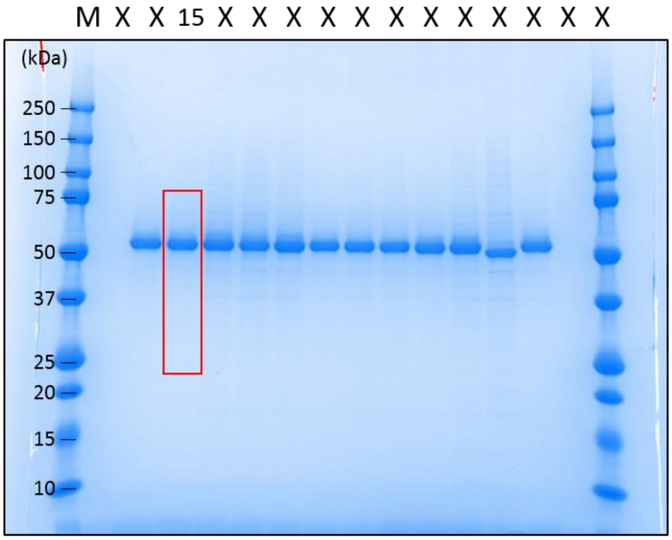

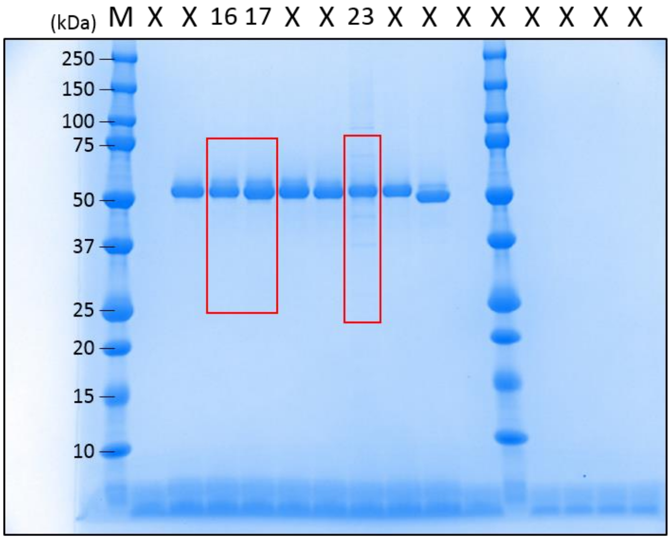

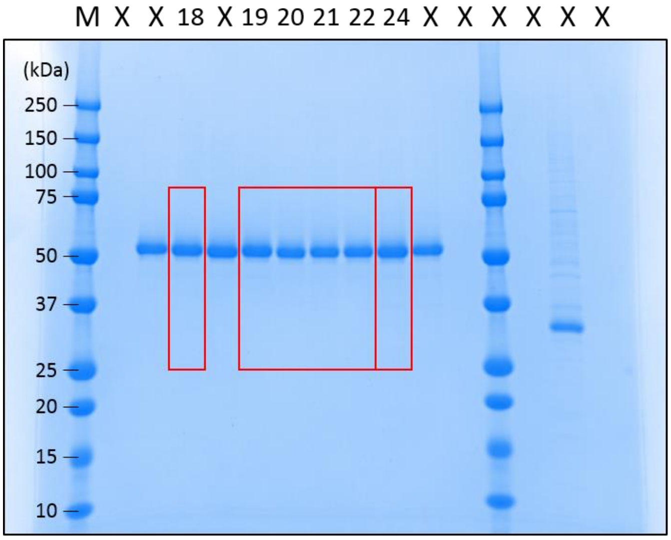

M. Precision Plus Protein Dual Color Standards (Bio-Rad, 161-0374)

1. pro-EK-MMP-9_Cat
2. pro-EK-MMP-9_Cat_F110A
3. pro-EK-MMP-9_Cat_Y179A
4. pro-EK-MMP-9_Cat_D185A
5. pro-EK-MMP-9_Cat_G186A
6. pro-EK-MMP-9_Cat_L187A
7. pro-EK-MMP-9_Cat_L188A
8. pro-EK-MMP-9_Cat_F192A
9. pro-EK-MMP-9_Cat_Y393A
10. pro-EK-MMP-9_Cat_L397A
11. pro-EK-MMP-9_Cat_V398A
12. pro-EK-MMP-9_Cat_L418A
13. pro-EK-MMP-9_Cat_P421A
14. pro-EK-MMP-9_Cat_Y423A
15. pro-EK-MMP-9_Cat_Q108N
16. pro-EK-MMP-9_Cat_T109F
17. pro-EK-MMP-9_Cat_E111P
18. pro-EK-MMP-9_Cat_P193A
19. pro-EK-MMP-9_Cat_I198V
20. pro-EK-MMP-9_Cat_Q199G
21. pro-EK-MMP-9_Cat_D410E
22. pro-EK-MMP-9_Cat_S413Q
23. pro-EK-MMP-9_Cat_Y420A
24. pro-EK-MMP-9_Cat_M422I

Raw image for S5B Fig: Purification and activation of MMP-9 mutants.

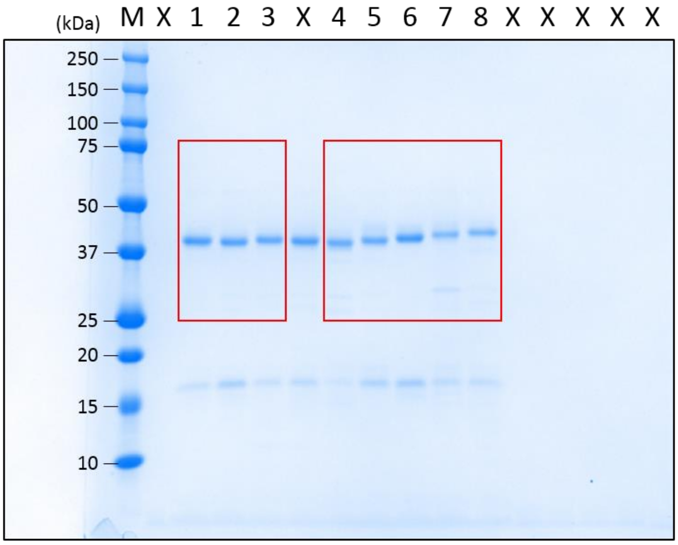

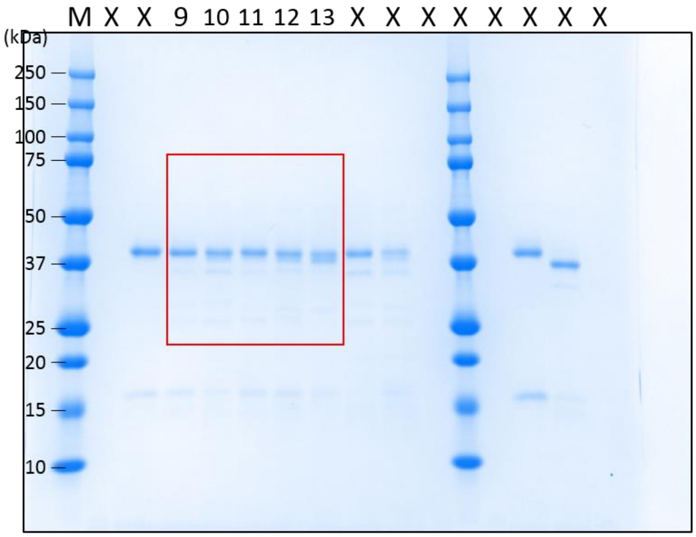

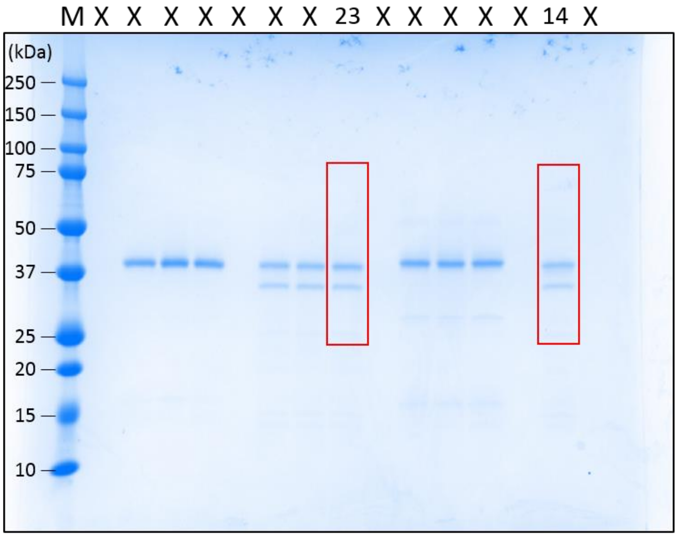

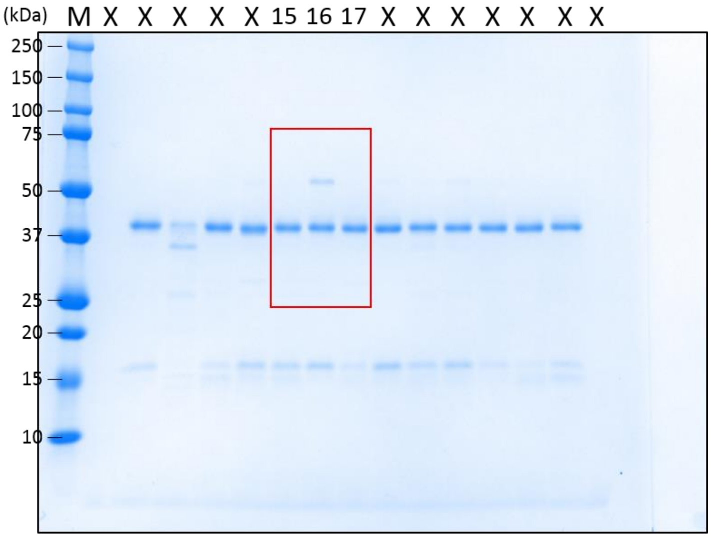

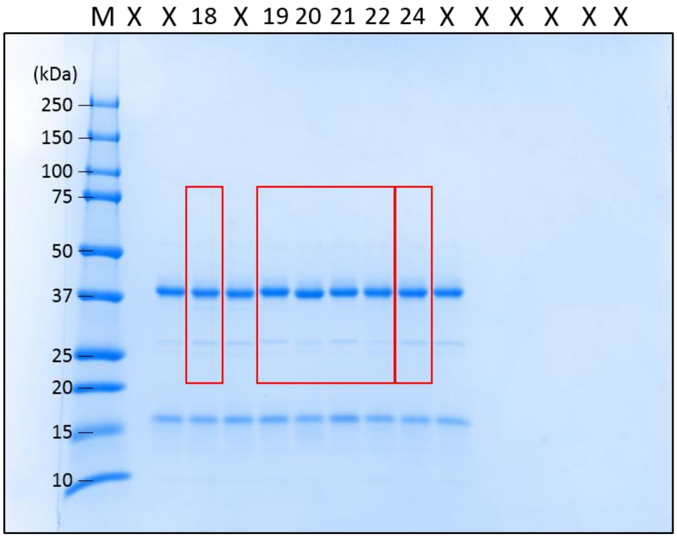

M. Precision Plus Protein Dual Color Standards (Bio-Rad, 161-0374)

1. active MMP-9_Cat
2. active MMP-9_Cat_F110A
3. active MMP-9_Cat_Y179A
4. active MMP-9_Cat_D185A
5. active MMP-9_Cat_G186A
6. active MMP-9_Cat_L187A
7. active MMP-9_Cat_L188A
8. active MMP-9_Cat_F192A
9. active MMP-9_Cat_Y393A
10. active MMP-9_Cat_L397A
11. active MMP-9_Cat_V398A
12. active MMP-9_Cat_L418A
13. active MMP-9_Cat_P421A
14. active MMP-9_Cat_Y423A
15. active MMP-9_Cat_Q108N
16. active MMP-9_Cat_T109F
17. active MMP-9_Cat_E111P
18. active MMP-9_Cat_P193A
19. active MMP-9_Cat_I198V
20. active MMP-9_Cat_Q199G
21. active MMP-9_Cat_D410E
22. active MMP-9_Cat_S413Q
23. active MMP-9_Cat_Y420A
24. active MMP-9_Cat_M422I

Raw image for S8 Fig: Expression of active-site mutants of MMP-9 (H401A, H405A, and H411A).

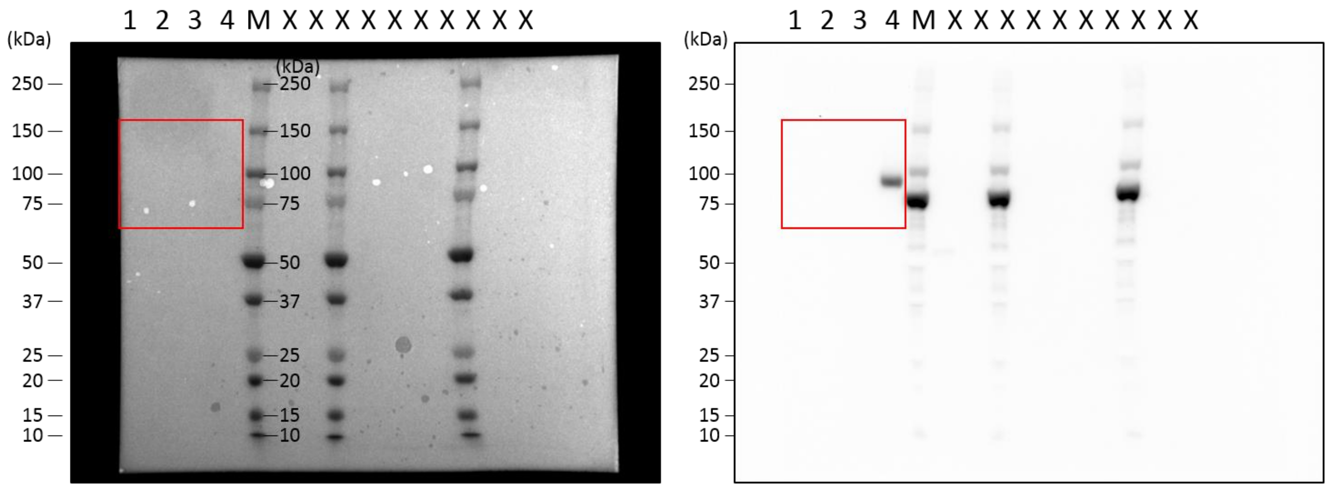

1. full-length MMP-9 H401A mutant fused to a C-terminal His_6 tag
2. full-length MMP-9 H405A mutant fused to a C-terminal His_6 tag
3. full-length MMP-9 H411A mutant fused to a C-terminal His_6 tag
4. full-length MMP-9 WT fused to a C-terminal His_6 tag

M. Precision Plus Protein Dual Color Standards (Bio-Rad, 161-0374)

Left, visible image of pre-stained protein marker
Right, chemiluminescent signal (Penta His HRP Conjugate, exposure time: 35 sec)

Raw image for S9 Fig: C-terminal degradation of MMP-9 during activation reaction using trypsin.

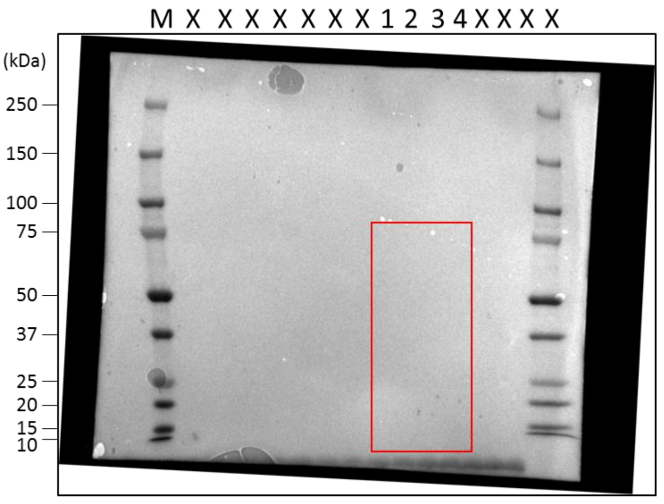

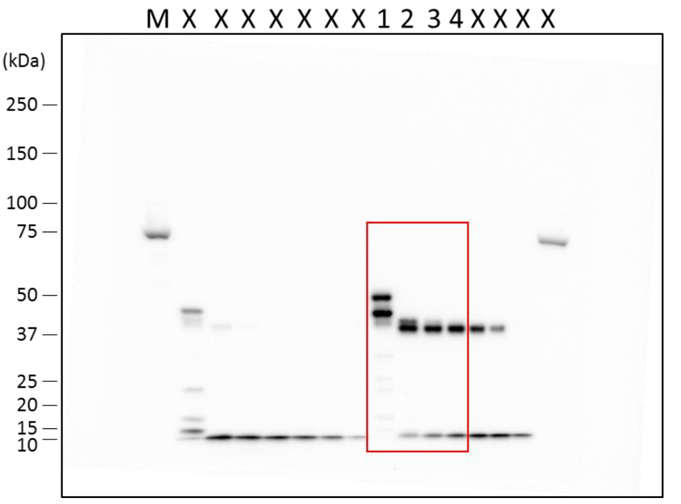

M. Precision Plus Protein Dual Color Standards (Bio-Rad, 161-0374)
1. activated pro-MMP-9_Cat-H6 by using trypsin, 0 h
2. activated pro-MMP-9_Cat-H6 by using trypsin, 1 h
3. activated pro-MMP-9_Cat-H6 by using trypsin, 2 h
4. activated pro-MMP-9_Cat-H6 by using trypsin, 3 h

Left, visible image of pre-stained protein marker
Right, chemiluminescent signal (Penta His HRP Conjugate, exposure time: 70 sec)

Raw image for S10 Fig: Autolysis of active MMP-9 upon long-term incubation at 25°C.

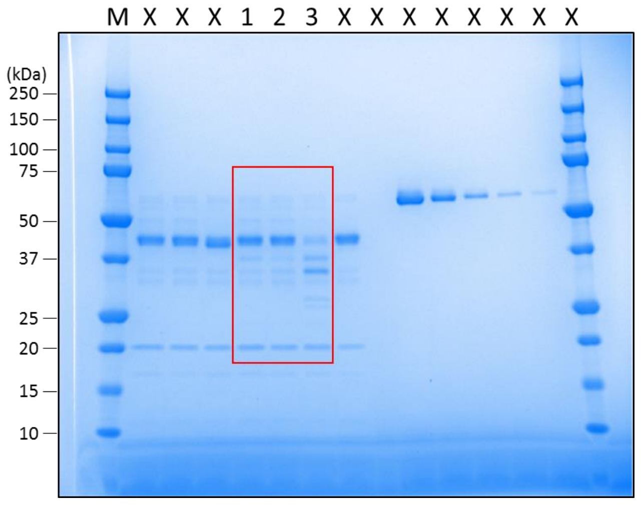

M. Precision Plus Protein Dual Color Standards (Bio-Rad, 161-0374)
1. biotinylated active MMP-9_Cat-BAP, -80°C, 16 h
2. biotinylated active MMP-9_Cat-BAP, 4°C, 16 h
3. biotinylated active MMP-9_Cat-BAP, 25°C, 16 h
